# Structural insights into the mechanism of a novel protein targeting pathway in Gram‐negative bacteria

**DOI:** 10.1002/2211-5463.12813

**Published:** 2020-03-09

**Authors:** Feng Jin

**Affiliations:** ^1^ School of Life Sciences Peking University Beijing China; ^2^ Peking‐Tsinghua Center for Life Sciences Peking University Beijing China

**Keywords:** Gram‐negative bacteria, OMPs, protein modeling, protein translocation, SecA^N^, supercomplex

## Abstract

Many nascent polypeptides synthesized in the cytoplasm are translocated across membranes via a specific ‘translocon’ composed of protein complexes. Recently, a novel targeting pathway for the outer membrane β‐barrel proteins (OMPs) in Gram‐negative bacteria was discovered. The cell envelope of Gram‐negative bacteria is composed of the inner (plasma) membrane (IM) and the outer membrane (OM). In this new pathway, a SecA^N^ protein, which is mainly present in the IM as a homo‐oligomer, translocates nascent OMPs across the IM; at the same time, SecA^N^ directly interacts with the β‐barrel assembly machinery (BAM) complex embedded within the OM. A supercomplex (containing SecA^N^, the BAM complex and many other proteins) spans the IM and OM, and is involved in the biogenesis of OMPs. Investigation of the function of SecA^N^ and the supercomplex, as well as the translocation mechanism, will require elucidation of their structures. However, no such structures are available. Therefore, here, I describe the use of protein modeling to build homology models for SecA^N^ and theoretical structures for the core‐complex composed of SecA^N^ and the BAM complex, which is a key part of the supercomplex. The modeling data are consistent with previous experimental observations and demonstrated a conformational change of the core‐complex. I conclude by proposing mechanisms for how SecA^N^ and the supercomplex function in the biogenesis of OMPs.

## Graphical abstract

I present here theoretical structures for the core part of a supercomplex that spans the inner and the outer membranes, and mediates the biogenesis of outer membrane β‐barrel proteins in Gram‐negative bacteria. This core‐complex, composed of the inner membrane protein translocation channel (SecA^N^) and the outer membrane β‐barrel assembly machinery, adopts different conformations, indicative of mechanisms for this new protein targeting pathway.

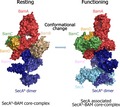

**Abbreviations**
BAM: β‐barrel assembly machinery
IM: inner (plasma) membrane
OM: outer membrane
OMP: outer membrane β‐barrel protein
POTRA: polypeptide transport‐associated
RMSD: root‐mean‐square deviation

Many proteins, synthesized by the cytosolic ribosomes [1, 2], will be guided by their intrinsic signals [3] and targeted to specific subcellular locations outside the cytoplasm via particular pathways [2, 4, 5]. During these processes, the newly synthesized polypeptides shall be translocated across the tightly sealed membranes through a specific ‘translocon’ that is usually composed of multiple proteins or protein complexes [4, 5, 6, 7, 8]. Many protein targeting pathways and translocons for various types of client protein [2, 5, 8, 9, 10, 11] have been identified, for example, the evolutionarily conserved *sec* pathway [12, 13]. In prokaryotic cells, the core component of the *sec* translocon is the membrane‐embedded heterotrimeric SecYEG protein complex, which forms the protein‐conducting channel (protein translocation channel) in the membrane [5, 10, 12, 13, 14, 15]. Meanwhile, either the translating ribosome or the substrate‐bound SecA protein (an ATPase) [16] could be associated with SecYEG at the cytoplasmic side, to deliver client proteins to the protein‐conducting channel in the translocon and to promote the translocation by either the elongation of the translating polypeptide or ATP hydrolysis [13, 17, 18, 19]. Most proteins, both membrane proteins and secreted proteins, were reported to be translocated by the *sec* translocon and through the SecYEG channel [12, 13]. In addition, new targeting pathways were identified in recent years [11, 20, 21, 22, 23].

A new targeting pathway for the outer membrane β‐barrel proteins (OMPs) in the Gram‐ negative bacteria was revealed recently [22, 23]. The envelope of Gram‐negative bacteria contains two membranes, the inner (plasma) membrane (IM) and the outer membrane (OM) [24]. The space between the IM and the OM is called the *periplasm* [24]. The OMPs are composed of β‐sheets and adopt an unusually cylindrical barrel‐like topology [25, 26]. In the currently prevailing model, the OMPs are synthesized in the cytoplasm and are delivered to the *sec* translocon aided by cytoplasmic chaperones such as the SecB protein [27, 28, 29]. Then, the OMPs are believed to be translocated across the IM through the SecYEG channel [27, 28, 29]. Subsequently, they are escorted by periplasmic chaperones to the OM and are finally integrated in the OM by the β‐barrel assembly machinery (BAM) complex, which is located in the OM to facilitate the folding and membrane insertion of OMPs [27, 28, 30, 31, 32, 33]. The *sec* translocon is supposed to export nascent OMPs in a ‘lateral gate’ mechanism [18, 19]. The SecA protein accepts nascent OMPs (which are in a precursor form and contain a signal peptide at the amino‐terminal) from SecB and transfers them into the SecYEG channel [29]. The binding of SecA to SecYEG and the insertion of signal peptides into the ‘lateral gate’ of the SecYEG channel will open the central pore of SecY [29]. SecA pushes the rest regions (mature regions) of OMPs passing through the pore [29], but previous experiments also demonstrated that, without the essential SecY [23, 34] or SecE [35] subunit, the translocation of OMPs could still occur.

In the new targeting pathway for OMPs that was identified in my previous work [22, 23], the SecA^N^ protein (a shortened form of the SecA protein, solely contains the N‐terminal region of SecA), existing as homo‐oligomers, was revealed to function in the IM for translocating OMPs and to directly interact with the essential BAM complex [23]. Furthermore, my colleagues and I unveiled that a protein supercomplex containing SecA, SecA^N^, SecYEG, the BAM complex and so forth spans the IM and the OM to meditate the biogenesis of OMPs [22]. According to these observations, we proposed a new model [22, 23]. In brief, in this model, SecA^N^ mainly forms the channel for the translocation of nascent OMPs and directly interacts with the nascent OMPs at either the signal peptide or the mature region [23]. SecA^N^ functions downstream SecA and may accept the nascent OMPs from SecA [23]. The translocation, folding and membrane insertion of OMPs were coordinated by the supercomplex [22, 23]. However, the mechanisms for SecA^N^ and the supercomplex were still not clear.

To further investigate how SecA^N^ and the supercomplex behave in the biogenesis of OMPs, structures of SecA^N^ and the supercomplex are required but have not been experimentally determined. The experimental determination of protein structures, especially of membrane protein structures, is usually a challenging task that requires sufficient expertise and resources and is time consuming. However, protein modeling is faster, and it is easier to predict either a protein’s tertiary structure according to its primary structure or the conformation of docked protein complexes, analyzing their behavior and revealing the mechanism, thus being adopted in this article. Homology models for protein sequences could be built using scientific algorithms provided by a Modeler program package (9v4) installed in the Discovery Studio^®^ Software, in which the protein sequence alignment, structure alignment, sequence similarity searching, model generation and model refinements were all included. The Modeler algorithm was primarily developed by Šali and Blundell [36] and commonly used to build the three‐dimensional (3D) structure for a protein sequence based on the known structures of its homologues. A ZDOCK algorithm could be applied to predict the structure of a protein complex. The ZDOCK algorithm was primarily established by Chen and Weng [37] to do the unbound protein–protein docking of two protein structures either experimentally determined or computationally modeled, using the Pairwise Shape Complementarity method [38]. Information about the binding sites could be input but is not essential when this program is run. The poses are clustered according to the ligand position and could be filtered through setting the binding site residues by users, according to experimental data or rational analysis.

In this study, first, the homology models for SecA^N^/SecA truncation/SecA were built via the Modeler package. Second, structures for the dimer of SecA^N^/SecA truncations/SecA mediated by the conserved GXXXG motif [23] were predicted through the ZDOCK algorithm. Third, mutants of SecA^N^/SecA truncation/SecA carrying mutations in the GXXXG motif were constructed using the Modeler package, to study the influences of these mutations on the structure, stability and dimer formation and to interpret previous observations [23]. Fourth, the models for the SecA^N^ dimer were docked to the experimentally determined structure of the BAM complex in either the ‘close’ or the ‘open’ conformation to reveal how SecA^N^ dimers interact with the BAM complex and to derive models for this SecA^N^‐BAM core‐complex (which is a key part of the supercomplex) based on experimentally identified binding site residues. Fifth, the models for the dimer of SecA truncation/SecA were docked to the BAM complex in either the ‘close’ or the ‘open’ conformation, to demonstrate that the regions that were not contained in SecA^N^ interfered with the interaction between the dimer of SecA truncation/SecA and the BAM complex. Sixth, the SecA^N^‐BAM core‐complex was unveiled to undergo a conformational change when it functioned in the biogenesis of OMPs, which furthermore resulted in proposing the possible mechanism for the supercomplex.

## Results

### Homology models for SecA^N^/SecA truncations/SecA were built using the Modeler program package

It has been revealed in a previous article [23] that a SecA^N^ protein, existing as homo‐oligomers in the IM of Gram‐negative bacteria, could translocate nascent OMPs across the IM (from the cytoplasm to the periplasmic space). Because no experimentally determined structure for SecA^N^ is available, the homology models of SecA^N^ were built in this article according to available structures of the full‐length SecA and other related ATPase proteins (Table S1). Based on the experimentally identified apparent molecular mass of SecA^N^ (~ 45 kDa) and the results of mass spectrometry analysis [23], the possible ending site of SecA^N^ may be around the residue position 400. Because the SecA protein contains multiple domains, including the nucleotide binding domain (NBD1 and NBD2), the protein binding domain (PPXD), the helical scaffold domain (HSD), the helical wing domain (HWD) and the Zn^2+^ binding regions, as shown in Fig. S1, the integrity of domains was considered as well. According to these, two possible ending sites for SecA^N^ are the residue 375 (containing the N‐terminal region of the NBD1 domain and the entire PPXD domain of SecA, the calculated molecular mass was ~ 42 kDa) and the residue 416 (containing the entire NBD1 and PPXD domain of SecA, ~47 kDa). Moreover, for comparison, homology models for the SecA596 truncation (ended at the residue 596, containing the NBD1, PPXD and NBD2 domains, ~66 kDa) were also built. It should be pointed out that structures of SecA proteins from different species and in different conformations have been experimentally determined in previous research, for example, the SecA structures listed in Table S1. However, a few regions of SecA were missing in these structures. Therefore, SecA homology models were also built and compared with the homology models of SecA^N^/SecA truncation to reveal whether and how the structure would be influenced when the SecA protein becomes shortened.

**Fig. 1:**
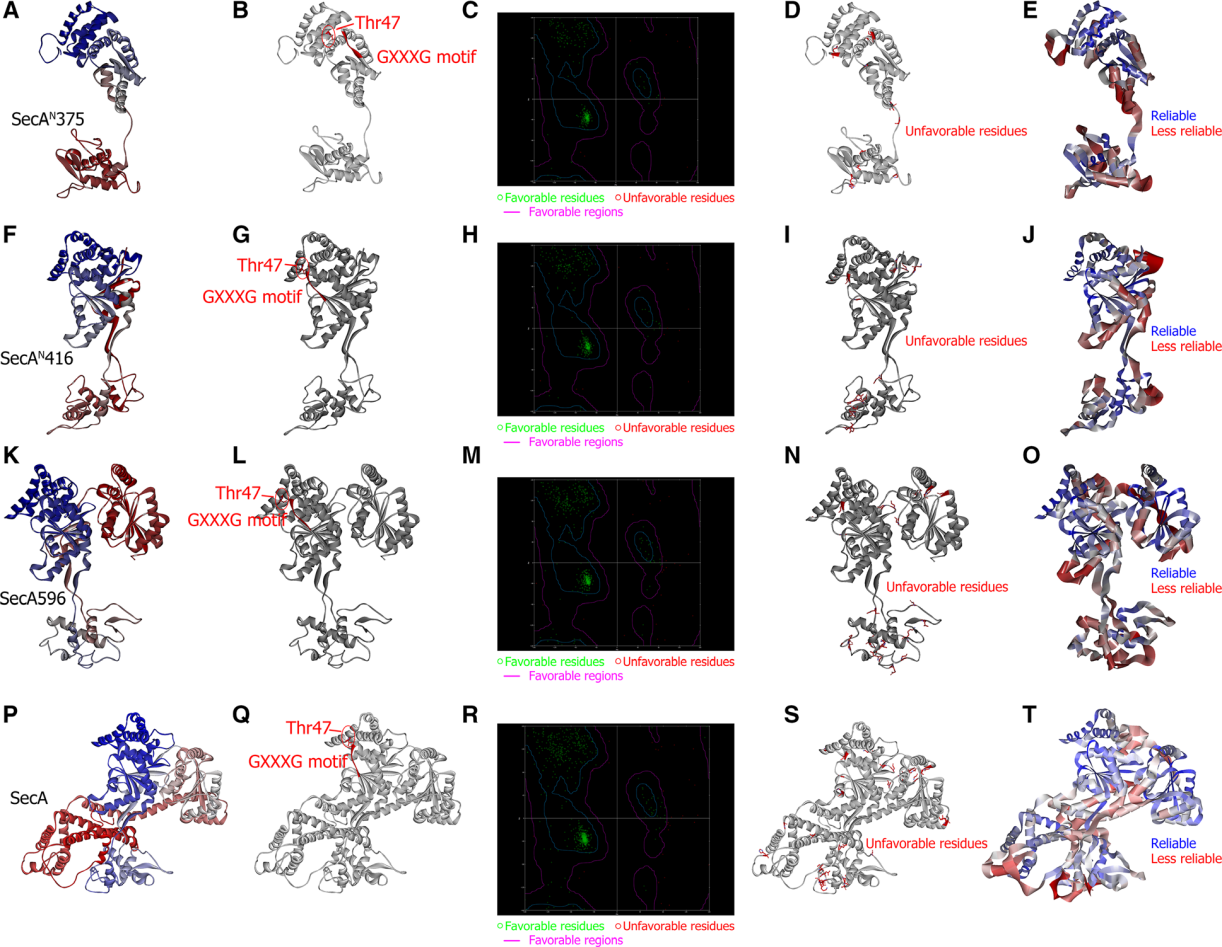
Homology models for SecA^N^/SecA truncation/SecA. (A, F, K, P) Shown are homology models for SecA^N^375 (A), SecA^N^416 (F), SecA596 (K) and SecA (P) generated with the Modeler algorithm. (B, G, L, Q) Shown are the positions of the GXXXG motif (colored red) and the residue 47 (shown as stick and colored red) in the homology models for SecA^N^375 (B), SecA^N^416 (G), SecA596 (L) or SecA (Q). (C, H, M, R) The Ramachandran plot for homology models of SecA^N^375 (C), SecA^N^416 (H), SecA596 (M) and SecA (R) were made to evaluate these models, in which favorable residues were represented by green points, whereas unfavorable residues were represented by red points. The purple line indicated the boundary of favorable regions. (D, I, N, S) The residues located in the unfavorable region of the Ramachandran plot were shown as stick and colored red in the homology models for SecA^N^375 (D), SecA^N^416 (I), SecA596 (N) and SecA (S). (E, J, O, T) Shown are verifications of homology models for SecA^N^375 (E), SecA^N^416 (J), SecA596 (O) and SecA (T) with the Profiles‐3D, in which the reliable regions were shown in narrow bands and colored blue, whereas the less reliable regions were shown in broad bands and colored red.

The sequences of SecA^N^/SecA truncation/SecA were input to search for related proteins that have 3D structures in the PDB_nr95 database via a ‘BLAST Search’ protocol. The structures of 10 proteins among the matched ones were selected as templates (Table S1), including five SecA proteins from different species and five other ATPase proteins. These templates were aligned to demonstrate their similarities. The main‐chain root‐mean‐square deviation (RMSD) and the number of overlapping residues are listed in Table S1. SecA protein structures 2IPC and 1NKT were the most similar, between which the number of overlapped residues were 791 and the main‐chain RMSD was 2.547 Å. Then, these templates were input to build homology models for SecA^N^/SecA truncation/SecA. In total, five models for each sequence were generated and sorted by the PDF Total Energy that is the sum of the scoring function value as listed in Table S2, among which the one with the lowest PDF Total Energy was optimal. Therefore, SecA^N^375_02 (Table S2A), SecA^N^416_02 (Table S2B), SecA596_01 (Table S2C) and SecA_05 (Table S2D), designated as SecA^N^375, SecA^N^416, SecA596 and SecA hitherto, were selected and used in the following modeling processes. These four models are shown in Fig. 1A,F,K,P. The highly conserved GXXXG motif that was revealed to be important for the dimer formation [23] was colored red; meanwhile, the residue 47, which has been cross‐linked with the BamA subunit of the BAM complex [23], was shown as stick and colored red (Fig. 1B,G,L,Q).

These predicted structures for SecA^N^/SecA truncation/SecA shown in Fig. 1A,F,K,P were checked with the Ramachandran plot [39], which showed the local backbone conformation of each residue in the model and indicated the favorable and unfavorable residues. The purple line was the boundary for favorable regions. As shown in Fig. 1C,H,M,R, most of the residues (represented by green points) were in the favorable region, and a few (represented by red points) were in the unfavorable region. As displayed in Fig. 1D,I,N,S, unfavorable residues, which are colored red and shown as sticks, were located in the loops of SecA^N^/SecA truncation/SecA. Then these models for SecA^N^/SecA truncation/SecA were verified with the Profiles‐3D, which was developed by D. Eisenberg’s group and used to check the validity of a theoretical protein structure by measuring the compatibility of that structure with the sequence of the protein [40]. As shown in Fig. 1E,J,O,T, the N termini, C termini, loops and few β‐strands (red and broad band) of SecA^N^/SecA truncation/SecA were less reliable than most of the helices (blue and narrow band). The Expected Low Score, Expected High Score and Verify Score are listed in Table S3. The Verify Scores for SecA^N^375, SecA^N^416, SecA596 and SecA were 103.43, 149.92, 211.64 and 334.71. Each of them was between the Expected Low Score and the Expected High Score. These results indicated that the models were mostly correctly built.

These four predicted structures of SecA^N^/SecA truncation/SecA shown in Fig. 1A,F,K,P were aligned with structures of the templates to study their similarities. The main‐chain RMSD and the number of overlapping residues are listed in Table S1. In particular, the alignments of shortened SecA (SecA^N^/SecA truncation) structures and indicated SecA structures are shown in Fig. S2, in which not the entire structure of SecA but solely the regions that were contained in the shortened SecA are displayed. These results indicated that SecA^N^375 and SecA were more similar to the SecA structure 1TF5, whereas SecA^N^416 or SecA596 was more similar to the SecA structure 3DIN or 2IPC, respectively. Furthermore, I aligned the structures of SecA^N^/SecA truncation/SecA with those of the SecA protein from *Escherichia coli* (2VDA and 2FSF) to compare the similarities of each domain. The main‐chain RMSD and the number of overlapping residues between NBD1(N) domains, PPXD domains, NBD1(C) domains, NBD2 domains and C‐terminal regions of these structures are listed in Table S4A–E, respectively. Among these domains, the NBD1 domains of these structures were more similar (Table S4A,C), whereas the PPXD domains shared the least similarity (Table S4B; 2FSF was not listed because a large amount of residues in the PPXD domain of 2FSF was missing and it was unable to do the alignment). In particular, the main‐chain RMSD between the PPXD domain of SecA^N^ and that of the full‐length SecA was extremely high (Table S4B). These results demonstrated that the structure of the PPXD domain was changed more than that of the other domains when the SecA protein became shortened. In addition, SecA^N^ did not contain the entire DEAD motor (composed of NBD1 and NBD2) domain. Therefore, SecA^N^ may not possess the ATPase activity and require the full‐length SecA to provide energy.

### SecA^N^ dimers mediated by the GXXXG motif were constructed using the ZDOCK algorithm

It has been revealed previously that SecA^N^ existed mainly as homo‐oligomers in the membrane, and the SecA^N^ dimer has been captured by photo‐cross‐linking experiments [23]. To predict the structure for the SecA^N^ dimer, I used a ZDOCK algorithm to address the initial stage of the SecA^N^‐SecA^N^ docking in this article. In this process, the available information could be used to block out residues from or force certain residues to be inside the binding interface. Previously, mainly via site‐specific mutagenesis, it was revealed that a highly conserved GXXXG motif (^151^GLTVG^155^), which has been commonly found in the transmembrane domain of membrane proteins and was involved in the membrane protein dimerization/oligomerization [41, 42], mediated the dimerization of SecA^N^ [23]. When the GXXXG motif of either the receptor or the ligand was forced to be inside the binding interface, in the filtered poses, Thr47 residues of both the receptor and the ligand were also very close to the binding interface, as shown in Fig. S3, for example. The residue 47 is located near the GXXXG motif, and it has been cross‐linked with BamA in living cells. However, in such structures (Fig. S3), the residue 47 of one SecA^N^ monomer was blocked by the other, and thus would be unable to interact with BamA. Therefore, it is more rational that solely one GXXXG motif is located in the binding interface. I then revised the parameters as follows: the receptor binding residues were the residues 151–155 (the GXXXG motif); the ligand‐blocked residue was the residue 47. Poses met with these criteria were filtered and clustered according to the position of the ligand. Then a 3D plot (Fig. S4) was made by plotting the ZDOCK score (*x*) versus the cluster (*y*) versus the density (*z*), to display all poses and to indicate ones with a high ZDOCK score and a high density. These poses were reranked with the ZRANK program [43], and ones with a ZRANK score higher than zero were not considered. Poses with a density lower than 2 and/or with a ZDOCK score lower than 15 were also eliminated. When all the poses in one cluster were eliminated, this cluster would be eliminated as well. From the first three clusters among the remaining ones, three representative models with a high ZDOCK score, a high density and a low ZRANK score were selected. These theoretical structures were then optimized with the Refine Docked Proteins (RDOCK) algorithm [44] to gain near‐native structures, which uses a CHARMm‐based procedure to carry out the two‐stage energy minimization.

For comparison, the models for the dimer of SecA truncation/SecA were also built in similar processes using identical parameters mentioned earlier. It should be pointed out that various crystal structures of SecA dimers in different conformations have been reported, demonstrating that there are various binding interfaces of the SecA dimer. But in none of them is the GXXXG motif located in the binding interface, indicating that the full‐length SecA may not use the GXXXG motif to form a dimer, or such type of the SecA dimer has not been obtained in experimental conditions. Therefore, to compare with the models for the dimer of SecA^N^/SecA truncation built in this article, I constructed models for the SecA dimer formed via the GXXXG motif using ZDOCK.

**Fig. 2:**
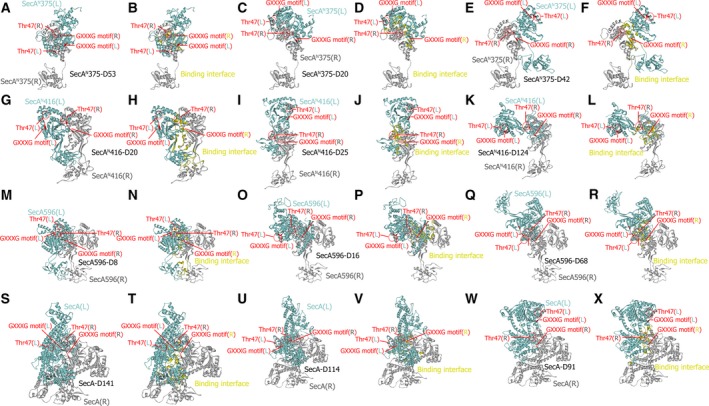
Theoretical structures for the dimer of SecA^N^/SecA truncation/SecA. (A, C, E) Shown are representative models for the SecA^N^375 dimer in different conformations, which are from pose 53, 20 and 42, and designated as SecA^N^375‐D53 (A), ‐D20 (C) and ‐D42 (E). (G, I, K) Shown are representative models for the SecA^N^416 dimer in different conformations, which are from pose 20, 25 and 124, and designated as SecA^N^416‐D20 (G), ‐D25 (I) and ‐D124 (K). (M, O, Q) Shown are representative models for the SecA596 dimer in different conformations, which are from pose 8, 16 and 68, and designated as SecA596‐D8 (M), ‐D16 (O) and ‐D68 (Q). (S, U, W) Shown are representative models for the SecA dimer in different conformations, which are from pose 141, 114 and 91, and designated as SecA‐D141 (S), ‐D114 (U) and ‐D91 (W). (B, D, F, H, J, L, N, P, R, T, V, X) The binding interface (colored yellow) for SecA^N^375‐D53 (B), ‐D20 (D), ‐D42 (F), SecA^N^416‐D20 (H), ‐D25 (J), ‐D124 (L), SecA596‐D8 (N), ‐D16 (P), ‐D68 (R), SecA‐D141 (T), ‐D114 (V) and ‐D91 (X) were demonstrated. In the models, the SecA^N^/SecA truncation/SecA monomer (the receptor) in which the GXXXG motif was inside the binding interface was colored gray; the other monomer (the ligand) in which the GXXXG motif was not inside the binding interface was colored cyan. The GXXXG motif was colored red, whereas the residue 47 was colored red and shown as stick.

The representative models for the dimer of SecA^N^/SecA truncation/SecA in different conformations are listed in Table S5 and shown in Fig. 2. The three theoretical structures for the SecA^N^375 dimer are shown in Fig. 2A,C,E, which were from pose 53, 20 and 42, respectively. They were designated hitherto as SecA^N^375‐D53, ‐D20 and ‐D42. Models for the dimer of SecA^N^416 (Fig. 2G,I,K), SecA596 (Fig. 2M,O,Q) and SecA (Fig. 2S,U,W) were displayed and designated (Table S5) as well. Key properties, including the density, cluster, ZDOCK score, ZRANK score and conformation of these models, are all listed in Table S5. In these models, residues in the GXXXG motif (colored red) of one monomer (the receptor; designated as SecA^N^375(R), SecA^N^416(R), SecA596(R) or SecA(R); colored light gray) directly interacted with the other monomer (the ligand; designated as SecA^N^375(L), SecA^N^416(L), SecA596(L) or SecA(L); colored cyan). The residue 47 (shown as stick and colored red) in the receptor was blocked by the ligand, whereas the residue 47 (shown as stick and colored red) in the ligand was exposed and located near one polar of the molecule, thus being proper for interacting with BamA in the periplasmic space. The binding interfaces for the dimer of SecA^N^375 (Fig. 2B,D,F), SecA^N^416 (Fig. 2H,J,L), SecA596 (Fig. 2N,P,R) and SecA (Fig. 2T,V,X) were marked yellow in the models.

### Mutations in the GXXXG motif interfered with the formation of SecA^N^ dimers

**Fig. 3:**
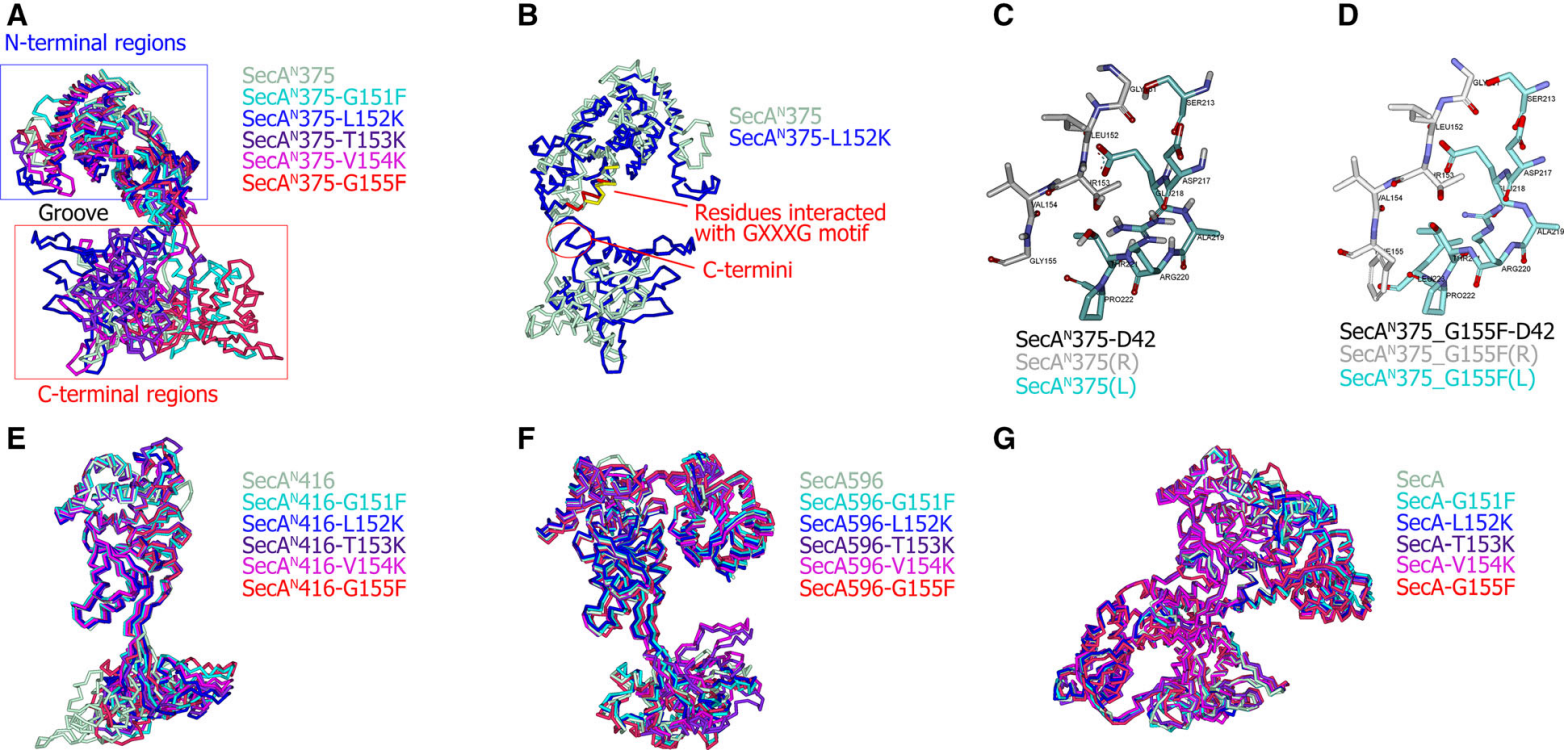
The influence of mutations in the GXXXG motif on the structure and dimer formation. (A) The homology models for SecA^N^375 and for the indicated SecA^N^375 mutants were aligned. The ‘Groove’ between the N‐ and C‐terminal was indicated. The conformational change of SecA^N^375‐L152K and SecA^N^375‐G155F mutants was larger than that of other mutants. (B) The SecA^N^375‐L152K mutant (blue) resulted in the C‐terminal regions being bent to the N‐terminal regions. Moreover, the C‐termini (red circle) became close to the residues that interacted with the GXXXG motif (colored yellow in SecA^N^375; colored red in SecA^N^375‐L152K) in SecA^N^375‐D42 and would interfere with the dimer formation. (C) Shown are the interaction between the GXXXG motif of the receptor (gray) and the residues 213 and 217–222 of the ligand (cyan) in SecA^N^375‐D42. (D) Through building the G155F mutation in SecA^N^375‐D42, the interference of this mutation on the interaction between the GXXXG motif of the receptor (carrying mutations, gray) and the residues 213 and 217–222 of the ligand (cyan) was shown. (E) The homology models for SecA^N^416 and for the indicated SecA^N^416 mutants were aligned. (F) The homology models for SecA596 and for the indicated SecA596 mutants were aligned. (G) The homology models for SecA and for the indicated SecA mutants were aligned. (A, B, E–G) SecA^N^375, SecA^N^416, SecA596 and SecA were colored light green, whereas G151F, L152K, T153K, V154K and G155F mutants were colored cyan, blue, purple, pink and red, respectively.

It was indicated in my previous study that mutating different residues in the GXXXG motif exerted different influences on the functions and dimer formation of SecA^N^ [23]. Therefore, homology models for mutants of SecA^N^/SecA truncation/SecA were built with the Modeler algorithm to investigate whether and how these mutations in the GXXXG motif affected the structure and/or stability of SecA^N^/SecA truncation/SecA. Templates (Table S1) and sequences carrying indicated mutations (Table S6) were input to generate homology models for mutants. For each mutant, five models were built, among which the one with the lowest PDF Total Energy was optimal and was used in the following processes. The PDF Total Energy, Verify Score and other properties for these optimal models are listed in Table S6, indicating that they were mostly correctly built. The energies of these models were calculated (Table S6) to evaluate their stabilities. These models for mutants were aligned with those for SecA^N^/SecA truncation/SecA to demonstrate influences of mutations on the structure (Fig. 3). The main‐chain RMSD and the number of overlapping residues are listed in Table S7. The influences of mutations in the GXXXG motif were classified into four types according to experimental observations and would be individually interpreted by the modeling data.

First, the previous study detected similar amounts of the SecA^N^‐L152K/SecA^N^‐G155F mutants and the wild‐type SecA^N^ protein, and similar amounts of the SecA‐L152K/SecA‐G155F mutants and the wild‐type SecA protein, when they were expressed from plasmids under the control of the natural promoter and regulator (*secM*) of the *secA* gene in *E. coli* cells [23]. Consistent with this, these two mutations did not affect much the stability of both SecA^N^375 (Table S6A) and SecA (Table S6D). A previous study also demonstrated that L152K and G155F mutations caused functional defects and interfered with the dimer formation of SecA^N^, but hardly exerted any influence on the function of the full‐length SecA [23]. Consistent with this, these two mutations affected the structure of SecA^N^375 (Fig. 3A) severely, but affected the structure of SecA (Fig. 3G) slightly. The main‐chain RMSD between the SecA^N^375‐L152K/SecA^N^375‐G155F mutant and SecA^N^375 was 5.907 Å/4.129 Å, which was much more than that (1.458 Å/2.192 Å) between the SecA‐L152K/SecA‐G155F mutant and SecA, as listed in Table S7A,D. The alignments of structures for SecA^N^375 and SecA^N^375 mutants are displayed in Fig. 3A, and those for SecA and SecA mutants are displayed in Fig. 3G. The ‘head’ (N‐terminal regions) of the SecA^N^375‐L152K mutant (blue) bent to its ‘tail’ (C‐terminal regions), whereas the ‘tail’ of the SecA^N^375‐G155F mutant (red) moved away from its ‘head’, indicating that the conformational change of these two mutants was larger than that of other mutants. The larger conformational change narrowed or broadened the ‘Groove’ between N‐ and C‐terminal regions more (Fig. 3A) and thus would interfere with the dimer formation, because in SecA^N^375‐D53 (Fig. 2A) and SecA^N^375‐D20 (Fig. 2C) the receptor just bound in the ‘Groove’ (Fig. 3A). In SecA^N^375‐D42 (Fig. 2E), the binding interface (Fig. 2F) was opposite to the ‘Groove’ but still in the region between the N‐ and C‐terminal that underwent a large conformational change (Fig. 3A). Moreover, as shown in Fig. 3B, the C termini (red circle) of the SecA^N^375‐L152K mutant became close to the residues 213 and 217–222 (colored yellow in SecA^N^375 or colored red in SecA^N^375‐L152K). These residues of the ligand interacted with the GXXXG motif of the receptor in SecA^N^375‐D42 (Fig. 2E), so the C termini (red circle) of the SecA^N^375‐L152K mutant interfered with the dimer formation. In addition, the Phe residue (Fig. 3D) was much larger than the Gly residue (Fig. 3C), so the G155F mutation would affect the interaction between the receptor (gray) and the ligand (cyan).

Second, the T153K mutation hardly affected the amount of SecA^N^ and SecA; meanwhile, it neither interfered with the dimer formation of SecA^N^ nor caused functional defects of both SecA^N^ and SecA [23]. Indeed, this mutation hardly affected the stability of SecA^N^375 (Table S6A) and SecA (Table S6D). It solely slightly changed their structures (Fig. 3A,G) as well. As listed in Table S7A,D, the main‐chain RMSD between SecA^N^375‐T153K and SecA^N^ was 2.772 Å, and that between SecA‐T153K and SecA was 1.348 Å.

Third, the G151F mutation reduced the amount and caused functional defects of SecA; moreover, it resulted in almost no detection of SecA^N^. This could also be interpreted by the modeling results. This mutation slightly influenced the structure of SecA^N^ (Table S7A) and SecA (Table S7D), and hardly affected the stability of SecA^N^ (Table S6A). But it caused extreme instability of SecA (Table S6D), which would reduce the amount of SecA; meanwhile, this would affect the function of SecA and the generation of SecA^N^ from the full‐length SecA.

Finally, no SecA^N^‐V154K and SecA‐V154K have been detected in the experiments [23]. Neither their stabilities (Table S6A,D) nor structures (Table S7A,D) were severely affected. I presumed that the mutation may change the secondary structure of the mRNA, thereby affecting the expression of these two mutants. The reason is worth further investigation.

These modeling data for SecA^N^375 and SecA could interpret my previous observations. However, the modeling data for SecA^N^416 were not consistent with the experimental results, because all these five mutations hardly affected both the stability (Table S6B) and the structure (Fig. 3E and Table S7B) of SecA^N^416. The modeling results for SecA596 were not consistent either, because the five mutations slightly affected the stability of SecA596 (Table S6C); moreover, their effects on the structure of SecA596 (Fig. 3F and Table S7C) even conflicted with the experimental results. Therefore, SecA^N^375 was more likely to approach to the native SecA^N^.

### The theoretical structures of the SecA^N^‐BAM core‐complex in different conformations were built using ZDOCK

In Gram‐negative bacteria, the folding and the membrane insertion of OMPs are facilitated by the BAM complex [30, 31, 32]. In *E. coli*, the BAM complex consists of five subunits, which are BamA, BamB, BamC, BamD and BamE [31, 32, 33]. The core subunit BamA is an OMP with its C‐terminal β‐barrel domain embedded in the OM and with its N‐terminal polypeptide transport‐associated (POTRA) domains (POTRA1–5) located in the periplasm [31, 32, 33]. The other four subunits are OM lipoproteins docked to the POTRA domains of BamA [31, 32, 33]. BamA is conserved in Gram‐negative bacteria, but the number of POTRA domains in BamA and the composition of the BAM complex vary in different species [45, 46]. Most bacteria BamA proteins contain five POTRA domains and in many bacteria (most Gama‐ and Beta‐proteobacteria), the BAM complex is composed of five subunits (BamA–E) [45, 46], which are similar to BamA and the BAM complex in *E. coli* [31, 32, 33]. The periplasmic regions of the BAM complex are large enough to approach the IM [22, 23]. Previous photo‐cross‐linking experiments demonstrated a direct interaction between SecA^N^ and BamA, and indicated that residues 121 and 129 of BamA (in the POTRA2 domain that is close to the IM), as well as the residue 47 of SecA^N^, were in the binding interface. So models for the SecA^N^ dimer (Fig. 2A,C,E,G,I,K) were docked to the experimentally determined structures of the BAM complex in either the ‘close’ (PDB: http://www.rcsb.org/pdb/search/structidSearch.do?structureId=5AYW) or the ‘open’ (PDB: http://www.rcsb.org/pdb/search/structidSearch.do?structureId=5EKQ) conformation, to predict how they interacted with each other and to construct models for this SecA^N^‐BAM core‐complex using ZDOCK. The experimentally identified binding site residues were forced to be inside the binding interface: the receptor (BAM) binding residues were the residues 121 and 129 of BamA; the ligand (the SecA^N^ dimer) binding residue was the residue 47 of SecA^N^375(L) or SecA^N^416(L). Besides, because SecA^N^ and the BAM complex are present in the IM and the OM, respectively, residues in the C‐terminal region of BamA, which are embedded in the OM, are unable to interact with SecA^N^. Therefore, the receptor‐blocked residues were those in the C‐terminal region (residue 424 to the end) of BamA. Poses met with these criteria were filtered. Then, 3D plots (Figs S5 and S6) were made to display poses and to select ones with a high ZDOCK score and a high density. Poses with a density lower than 2 and/or with a ZDOCK score lower than 15 were then eliminated. Moreover, poses in which the model adopted a wrong conformation were eliminated as well. In such models, one or both SecA^N^ monomers were almost parallel to the membrane. Considering the orientation of the BAM complex and the width of the periplasmic space, such SecA^N^ monomers were unable to be inserted in the IM but were located in the periplasmic space (Fig. S7). It was inconsistent with the experimental results that almost all the SecA^N^ proteins existed in the membrane [23]. The remaining poses were then refined with the RDOCK program.

**Fig. 4:**
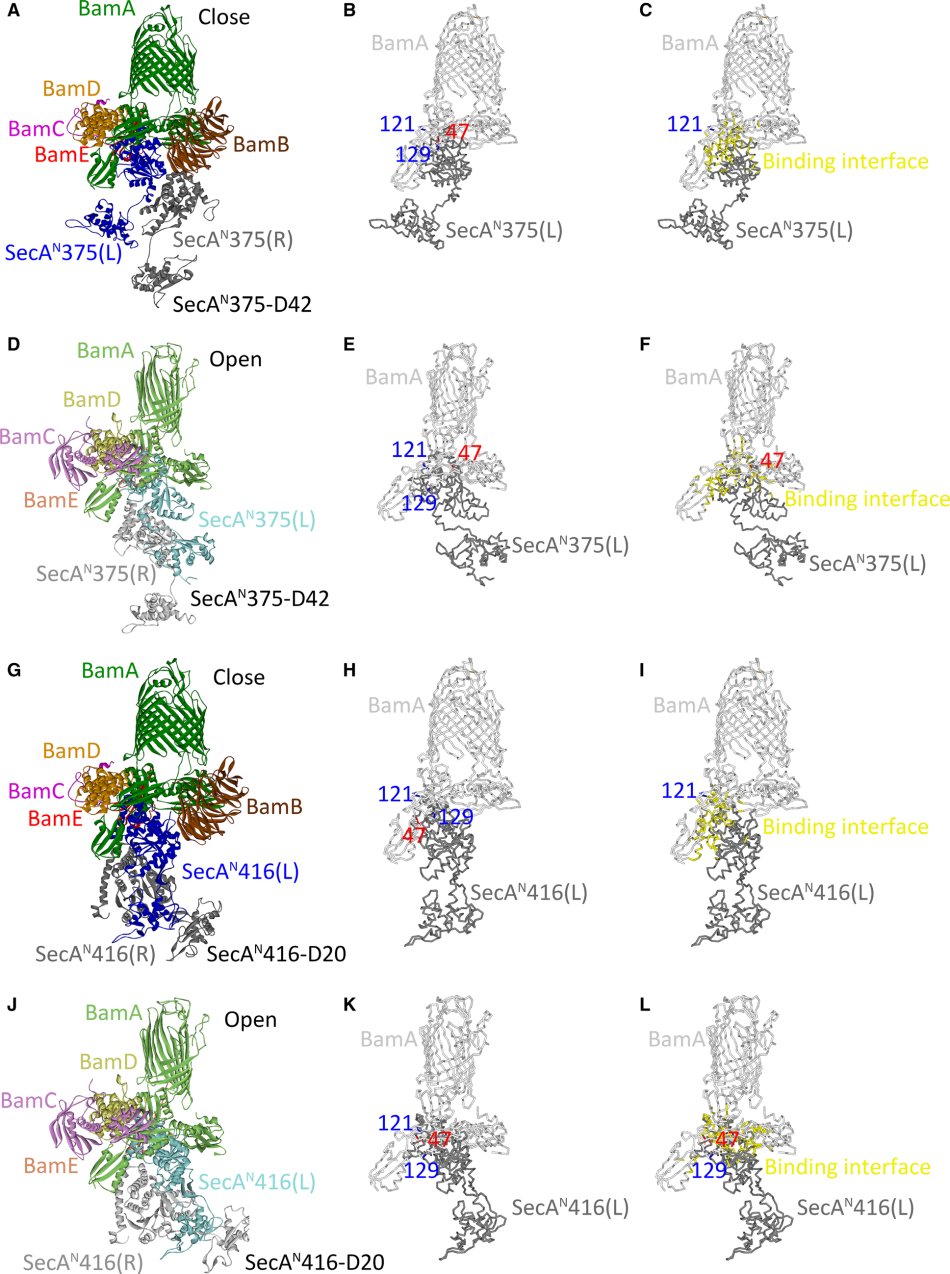
Theoretical structures for the SecA^N^375‐BAM core‐complex and the SecA^N^416‐BAM core‐complex in different conformations. (A, D, G, J) SecA^N^375‐D42 was docked to the BAM complex in either the ‘close’ conformation (A) or the ‘open’ conformation (D). SecA^N^416‐D20 was docked to the BAM complex in either the ‘close’ conformation (G) or the ‘open’ conformation (J) as well. (A, G) The subunits of the BAM complex were colored dark green (BamA), brown (BamB), purple (BamC), orange (BamD) and red (BamE). SecA^N^375(L) and SecA^N^416(L) were colored blue; meanwhile, SecA^N^375(R) and SecA^N^416(R) were colored dark gray. (D, J) The subunits of the BAM complex were colored light green (BamA), light purple (BamC), yellow (BamD) and vermilion (BamE). SecA^N^375(L) and SecA^N^416(L) were colored cyan; SecA^N^375(R) and SecA^N^416(R) were colored light gray. (B, E, H, K) Displayed were positions of the residue 121 and the residue 129 of BamA, which were shown as stick and colored blue, as well as the residue 47 of SecA^N^375(L) or SecA^N^416(L), which was shown as stick and colored red. Solely BamA (colored light gray) of the BAM complex and SecA^N^375(L) or SecA^N^416(L) (colored dark gray) were shown. The BAM complex was in the ‘close’ conformation in (B) and (H), but in the ‘open’ conformation in (E) and (K). (C, F, I, L) The binding interface between the SecA^N^ dimer and the BAM complex was marked yellow. BamA was colored light gray, and SecA^N^375(L) or SecA^N^416(L) was colored dark gray. The BAM complex was in a ‘close’ conformation in (C) and (I), but in an ‘open’ conformation in (F) and (L). Residues 121 and 129 of BamA or the residue 47 of SecA^N^ that were not in the binding interface were shown as stick and colored blue or red. The PDB number of the BAM complex in a ‘close’ conformation: http://www.rcsb.org/pdb/search/structidSearch.do?structureId=5AWY; the PDB number of the BAM complex in an ‘open’ conformation: http://www.rcsb.org/pdb/search/structidSearch.do?structureId=5EKQ.

Among the six models for the SecA^N^ dimer, SecA^N^375‐D42 and SecA^N^416‐D20 could form proper complexes with the BAM complex in either the ‘close’ or the ‘open’ conformation, indicating that the SecA^N^‐BAM core‐complex could adopt different conformations. The key properties for these models, including the poses, binding site residues (mainly focused on the experimentally identified binding site residues, which were the residues 121 and 129 of BamA and the residue 47 of SecA^N^), density, cluster, ZDOCK score and conformation, are listed in Table S8 (‘Y’ represented ‘yes’, indicating that the residue was in the binding interface; ‘W’ represented ‘wrong conformation’). Because solely when the BAM complex was in an ‘open’ conformation could models (such as pose 28 in Table S8B and pose 42 in Table S8D) in which the residue 121 was inside the binding interface be obtained, indicating that these models represented one state of the core‐complex that was designated as the ‘open’ conformation (Fig. 4D,J). To the contrary, when the BAM complex was in the ‘close’ conformation, representative models in Table S8A (such as pose 2) and Table S8C (such as pose 9) represented another state of the core‐complex (the residue 129 of BamA and the residue 47 of SecA^N^ were in the binding interface) that was designated as the ‘close’ conformation (Fig. 4A,G). These different conformations of the SecA^N^‐BAM core‐complex were captured by photo‐cross‐linking experiments but have not been distinguished [23]. With protein modeling, the structures for the core‐complex in different conformations were predicted.

Most of the models listed in Table S8 were similar to those shown in Fig. 4A,D,G,J, in which the SecA^N^ dimer was almost vertically inserted in the IM with its N‐terminal regions exposed to the periplasmic space and interacting with the BAM complex. The periplasm‐exposed regions of the SecA^N^ dimer bound to the POTRA domains of BamA and inserted in the center of the periplasmic region of the BAM complex. The SecA^N^ dimer also interacted with BamB and BamD, but solely in Fig. 4J did it interact with BamE. The SecA^N^ dimer faced to the channel of the barrel formed by the C‐terminal region of BamA in the OM. The SecA^N^ dimer did not completely block the channel from the periplasmic side in the ‘close’ conformation, whereas it did in the ‘open’ conformation. The five subunits of the BAM complex and the two SecA^N^ monomers were labeled and shown in different colors as indicated in Fig. 4. In Fig. 4B,C,E,F,H,I,K,L, solely BamA, as well as SecA^N^375(L) or SecA^N^416(L), is shown; the residues 121 and 129 of BamA and the residue 47 of SecA^N^ are labeled and shown in blue and red, respectively, as indicated in the figure. The binding interface was marked yellow as shown in Fig. 4C,F,I,L.

### The regions that were not contained in SecA^N^ interfered with the interaction between the dimer of SecA596/SecA and the BAM complex

**Fig. 5:**
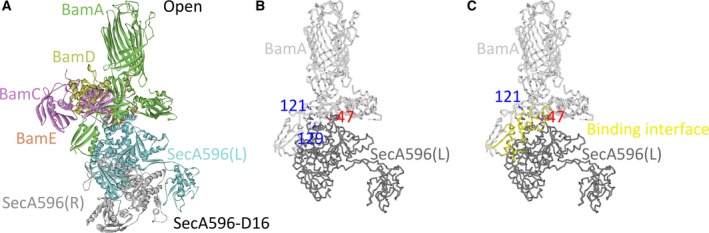
Models for the SecA596‐BAM core‐complex in the ‘open’ conformation. (A) SecA596‐D16 was docked to the BAM complex in the ‘open’ conformation. The subunits of the BAM complex were colored light green (BamA), light purple (BamC), yellow (BamD) and vermilion (BamE). SecA596(L) was colored cyan, and SecA596(R) was colored light gray. (B) Displayed were positions of the residues 121 and 129 of BamA, which were shown as stick and colored blue, as well as the residue 47 of SecA596(L), which was shown as stick and colored red. Solely BamA (colored light gray) of the BAM complex and SecA596(L) (colored dark gray) were shown. (C) The binding interface between the SecA596 dimer and the BAM complex was marked yellow. BamA was colored light gray, and SecA596(L) was colored dark gray. Residues 121 and 129 of BamA or the residue 47 of SecA^N^ that was not in the binding interface was shown as stick and colored blue or red. The PDB number of the BAM complex in an ‘open’ conformation: http://www.rcsb.org/pdb/search/structidSearch.do?structureId=5EKQ.

The models for either the SecA596 dimer (SecA596‐D8, ‐D16 and ‐D68) or the SecA dimer (SecA‐D141, ‐D114 and ‐D91) shown in Fig. 2M,O,Q,S,U,W were docked to the BAM complex in either the ‘close’ (PDB: http://www.rcsb.org/pdb/search/structidSearch.do?structureId=5AYW) or the ‘open’ (PDB: http://www.rcsb.org/pdb/search/structidSearch.do?structureId=5EKQ) conformation. Besides, the experimentally determined structure of the *E. coli* SecA dimer (PDB: http://www.rcsb.org/pdb/search/structidSearch.do?structureId=2FSF; Fig. S8) in which the GXXXG motif was not in the binding interface was also docked to the BAM complex for comparison. Still, residues 121 and 129 of BamA, as well as the residue 47 of SecA^N^, were set to be inside the binding interface, whereas the C‐terminal region of BamA was blocked from the interaction. The filtered poses are displayed in Figs S9 and S10. Solely SecA596‐D16 could form a complex with the BAM complex in the ‘open’ conformation (Table S9), but none of the models for the SecA596 dimer could form a proper complex with the BAM complex in the ‘close’ conformation. Models for the SecA dimer could not form a proper complex with the BAM complex in either the ‘close’ or the ‘open’ conformation. Because the N‐terminal regions of SecA596 and SecA were larger than those of SecA^N^, they would be hindered from being inserted in the center of the BAM complex, leading to the rotation of SecA596 or SecA from vertical to parallel, as displayed in Fig. 5A, for example. In this theoretical structure, SecA596‐D16 was docked to the BAM complex in the ‘open’ conformation, in which solely one SecA596 monomer directly interacted with the BAM complex, almost lying in the membrane. In Fig. 5B,C, solely BamA and SecA596(L) are shown; the residues 121 and 129 of BamA, as well as the residue 47 of SecA596, are labeled and shown in blue and red, respectively, as indicated in the figure. The binding interface was marked yellow as shown in Fig. 5C. These results indicated that the experimentally identified binding interface for the SecA^N^ dimer was not suitable for the SecA596 dimer or the SecA dimer, because the additional regions including NBD1(C), NBD2 and so forth that were not contained in SecA^N^ would interfere with the interaction between the dimer of SecA596/SecA and the BAM complex. However, these results did not completely exclude the opportunity for the full‐length SecA to directly interact with the BAM complex, because the full‐length SecA may interact with the BAM complex as a monomer or bind to other regions of the BAM complex.

## Discussion

In this article, I first built the homology models for SecA^N^/SecA truncation/SecA using the Modeler algorithm (Fig. 1 and Table S2). Then, I docked the models for SecA^N^/SecA truncation/SecA to predict the structure of their dimers mediated by the GXXXG motif (Fig. 2 and Table S5). Next, I constructed models for mutants of SecA^N^/SecA truncation/SecA carrying mutations in the GXXXG motif to study the influences of mutations on the stability (Table S6), structure (Fig. 3 and Table S7) and dimer formation (Fig. 3). Subsequently, I docked the models for the SecA^N^ dimer to the experimentally determined structure of the BAM complex in either the ‘close’ (PDB: http://www.rcsb.org/pdb/search/structidSearch.do?structureId=5AYW) or the ‘open’ (PDB: http://www.rcsb.org/pdb/search/structidSearch.do?structureId=5EKQ) conformation, to predict structures for the SecA^N^‐BAM core‐complex (Fig. 4 and Table S8). Finally, I docked the dimer of SecA truncation/SecA to the BAM complex in either the ‘close’ or the ‘open’ conformation (Fig. 5 and Table S9) but hardly obtained proper models, indicating the interference of regions that were not contained in SecA^N^ with the formation of such complexes.

**Fig. 6:**
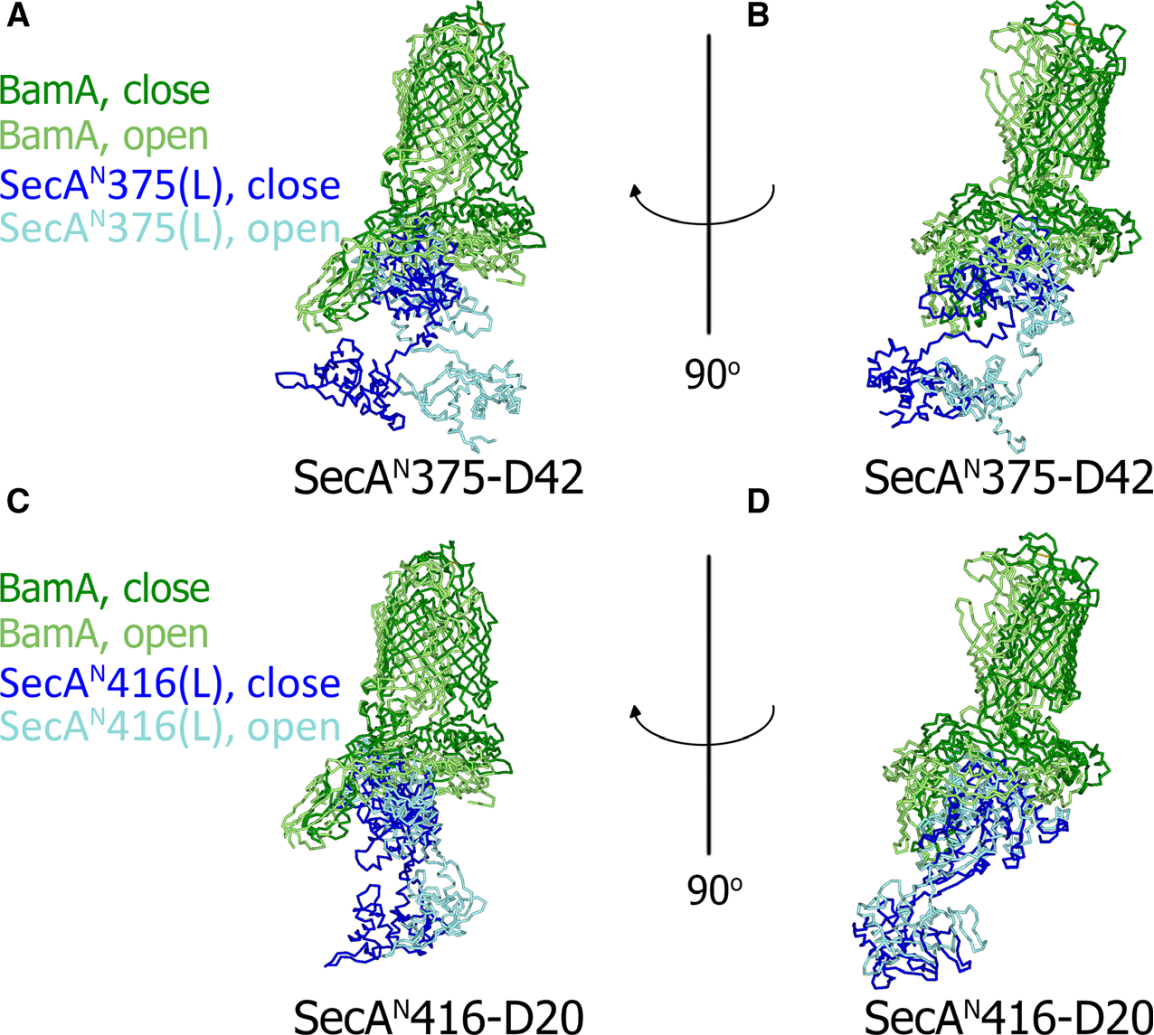
The conformational change of the SecA^N^‐BAM core‐complex. (A, B, C, D) Shown are the aligned structures of the SecA^N^375‐BAM core‐complex (A, B) in different conformations or the SecA^N^416‐BAM core‐complex (C, D) in different conformations. Solely the BamA subunit of the BAM complex and SecA^N^375(L)/SecA^N^416(L) were displayed. When the SecA^N^375‐BAM core‐complex was in the ‘close’ conformation, BamA was colored dark green and SecA^N^375(L) or SecA^N^416(L) was colored blue. When the SecA^N^375‐BAM core‐complex was in the ‘open’ conformation, BamA was colored light green and SecA^N^375(L) or SecA^N^416(L) was colored cyan. The structures in (A) or (C) were rotated 90° and shown in (B) or (D), respectively. The PDB number of the BAM complex in a ‘close’ conformation: http://www.rcsb.org/pdb/search/structidSearch.do?structureId=5AWY; the PDB number of the BAM complex in an ‘open’ conformation: http://www.rcsb.org/pdb/search/structidSearch.do?structureId=5EKQ.

Based on the earlier modeling data, mechanisms for the SecA^N^‐BAM core‐complex in the biogenesis of OMPs could be proposed. It is demonstrated in Fig. 4 and Table S8 that the core‐complex could adopt the ‘close’ and the ‘open’ conformations. The theoretical structures of the core‐complex in the ‘close’ conformation (Fig. 4A,G) were aligned with that in the ‘open’ conformation (Fig. 4D,J) to demonstrate how the conformation of the core‐complex was changed as shown in Fig. 6, in which solely the BamA subunit of the BAM complex and SecA^N^(L) were displayed. It was demonstrated that the SecA^N^375 dimer turned about 180° and rotated a large angle from the position in the ‘close’ conformation (colored blue) to the position in the ‘open’ conformation (colored cyan) as shown in Fig. 6A,B. However, as displayed in Fig. 6C,D, although the SecA^N^416 dimer also rotated when the conformation of the core‐complex changed from ‘close’ to ‘open’, the rotation angle was much smaller than that of the SecA^N^375 dimer (Fig. 6A,B), indicating that the relatively larger SecA^N^416 dimer was less flexible. The SecA^N^375 dimer bound to the POTRA domains of BamA (Fig. 6A,B). In the ‘close’ conformation, the SecA^N^375 dimer directly interacted with the POTRA1, 2, 3 and 5 of BamA. But in the ‘open’ conformation, the SecA^N^375 dimer turned and rotated to interact with all five of the POTRA domains.

**Fig. 7:**
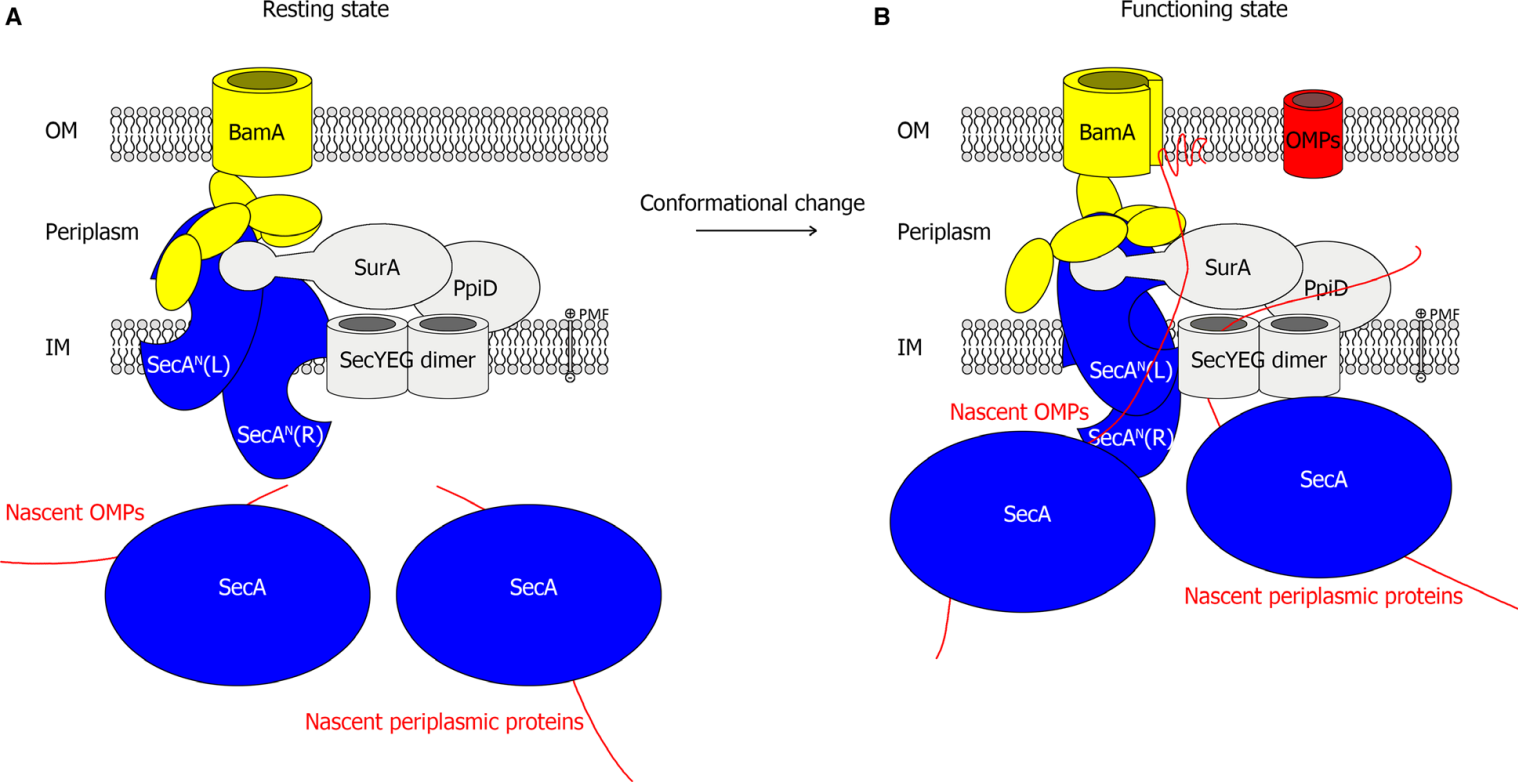
A schematic model for the supercomplex. Shown is a schematic model for the supercomplex that spans the IM and the OM to mediate the biogenesis of OMPs. SecA^N^ oligomers (indicated as a dimer, blue) form the channel for translocating OMPs across the IM, whereas the SecYEG dimer (gray) functions as a channel for the translocation of periplasmic proteins and so forth. The IM translocons are associated with the OM BAM complex (solely BamA were displayed and colored yellow). In the biogenesis of OMPs, the binding of SecA (blue) and nascent OMPs (red) drive the conformational change of the supercomplex from the ‘resting state’ (A) to the ‘functioning state’ (B). During this process, SecA^N^ turns about 180° and rotates, inducing the conformational change of the periplasmic domains of BamA and the lateral open of the barrel of BamA (B).

Because the SecA^N^‐BAM core‐complex is a key part of the supercomplex, the mechanism for the core‐complex could also infer how the supercomplex would function in the biogenesis of OMPs. In addition, a ‘lateral gate’ model has been proposed [33], in which the barrel of BamA is laterally opened during the biogenesis of OMPs. According to the previous model [33] and the theoretical structure for the core‐complex constructed in this article, the schematic model for the supercomplex is illustrated in Fig. 7 to discuss the possible mechanism. Although multiple conformations of the SecA^N^ dimer have been predicted, solely the modeling data (Figs 3 and 4 and Tables S6–S8) of SecA^N^375‐D42 were consistent with almost all of the experimental results that have been obtained so far [22, 23], indicating that it approaches the native conformation of the SecA^N^ dimer. So the schematic model was drawn according to the theoretical structure of the core‐complex constructed with SecA^N^375‐D42 and the BAM complex.

In this model (Fig. 7), a supercomplex [22, 23] mainly composed of SecA, SecA^N^, SecYEG, PpiD, SurA and the BAM complex, spanning the IM and the OM, is involved in the biogenesis of OMPs. The SecA^N^ oligomer (colored blue and indicated as a dimer in the figure) forms the channel for nascent OMPs in the IM and directly interacts with the BAM complex (solely BamA were displayed; colored yellow) to form a bridge across the IM and the OM. The SecYEG complex (colored gray and shown as a dimer) formed the channel for multiple client proteins, including many components of the supercomplex, for instance, the IM protein SecY, the periplasmic protein SurA, the lipoprotein PpiD, among others. The association of these two protein‐conducting channels may be convenient for the biogenesis and assembly of the supercomplex, because the supercomplex is composed of various types of proteins that respectively relied on the two channels for translocations. Meanwhile, it would be efficient for regulating the amount of client proteins (OMPs) and the amount of supercomplex components. The SecA protein (blue) could be present in the cytoplasm or associated with the membrane/translocon to transfer the nascent client proteins to the channels. Moreover, it might also be membrane integrated (data not shown), because a small amount of SecA has been reported to be permanently inserted in the membrane [47]. In the biogenesis of OMPs, the supercomplex is in the ‘resting state’ (Fig. 7A) when no client protein (nascent OMP) binds. However, when SecA delivers the nascent OMP to the supercomplex and is associated with it, the conformation of the supercomplex is changed to the ‘functioning state’ (Fig. 7B), resulting in the rotation of SecA^N^ and the lateral open of BamA. Simultaneously, the translocation of the nascent OMP across the IM to the periplasm is initiated. The newly translocated regions of the nascent OMP will be immediately transported, folded and inserted into the OM by the supercomplex before the translocation of the entire polypeptide is completed. In conclusion, the translocation, folding and membrane integration process during the biogenesis of OMPs are not isolated but coordinated by the supercomplex. This supercomplex undergoes a conformational change to facilitate the biogenesis of OMPs. Considering that in the supercomplex solely SecA could provide energy through ATP hydrolysis, the driving force for transporting clients and for the conformational change may be provided by SecA.

Some issues about this model are worth noting. The first issue is that SecA has been found to be a cytoplasmic protein that could be associated with SecYEG [19, 48], but SecA and particularly its shortened form SecA^N^ have some unique behaviors unlike those of a typical cytoplasmic protein. First, SecA could be associated to the membrane lacking SecYEG [27]. In addition, the conditions that could remove peripheral proteins were unable to extract all SecA and SecA^N^ proteins from the membrane; moreover, a portion of SecA and almost all of the SecA^N^ proteins are permanently inserted in the membrane [23, 47, 49]. Some regions of SecA and SecA^N^ could even be exposed to the periplasm [23, 50]. These results all indicated that SecA and SecA^N^ may be penetrated more deeply or even integrated into the membrane; therefore, the membrane‐inserted SecA^N^ is able to reach the large periplasmic region of the BAM complex. SecA^N^ and SecA are not typical integral membrane proteins, so it is presumed that the membrane insertion of SecA^N^ and SecA may be stabilized by their interactions with other components of the supercomplex, such as the BAM complex and SecYEG. The second issue is that photo‐cross‐linking experiments indicated that there is a direct interaction between SecA^N^ and BamA. Because BamA itself is an OMP, the translocation of nascent BamA may also rely on SecA, SecA^N^ and so forth. It is possible that nascent BamA is cross‐linked with these factors during its biogenesis. Therefore, to eliminate the photo‐cross‐linked products between nascent BamA and SecA^N^/SecA, I added chloramphenicol into the broth (in the midlog phase) to stop the protein synthesis in the cells. Photo‐cross‐linking experiments were performed about 20 min later, when BamA synthesis was ceased and almost no nascent BamA was being translocated, but SecA^N^ could still be cross‐linked with BamA (data not shown), indicating that they could interact with each other as functional partners. The third issue is that because there is no signal sequence in both SecA^N^ and SecA for secretion, how were they targeted to the membrane? According to previous reports, SecA and SecA^N^ could be targeted to the vicinity of the membrane/*sec* translocon via the expression regulation system of the *secA* gene [51]. The translation of *secA* gene is regulated in response to the secretion by a secreted SecM protein that is exported to the periplasm by the *sec* translocon [48, 51, 52]. The *secM* gene was in the same operon with the *secA* gene, but located upstream of it [51]. SecM translation is transiently arrested presumably because of an ‘arrest sequence’ at its C‐terminal region [52]. The stalled ribosome changes the secondary structure of the *secM*‐*secA* mRNA, resulting in the exposure of the Shine–Dalgarno sequence for the translation of *secA* [51]. With this mechanism, the translation of *secA* may occur quite close to the membrane/*sec* translocon, but how SecA^N^ is inserted into the membrane and assembled into the supercomplex to form a channel requires further investigation. The membrane‐insertion and assembly mechanism for some pore‐forming toxins [53] may shed light on the future study of this issue. The fourth issue is that in the previous work, my colleagues and I discovered that SecA functions upstream of SecA^N^ and may deliver the client proteins to SecA^N^ [22, 23]. Is it possible that SecA directly interacts with SecA^N^ as illustrated in the model? I docked the structure of SecA (Fig. 1P) to the model of the SecA^N^‐Bam core‐complex (Fig. 4A,D), and thus potential binding regions for SecA in the cytoplasmic side of SecA^N^ (C‐terminal regions) were indicated (Fig. S11). Besides, SecA could be associated with the core‐complex in the ‘open’ conformation (Fig. 4D), but no proper model was obtained when SecA was docked to the core‐complex in the ‘close’ conformation (Fig. 4A), further demonstrating that the supercomplex undergoes a conformational change after SecA binding.

## Materials and methods

### Homology models for SecA^N^/SecA truncation/SecA and their mutants were built using the Modeler package

Protein structures related to query sequences, including the sequences of SecA^N^/SecA truncation/SecA and their mutants, were searched in the PDB_nr95 database through the BLAST Search protocol. The BLAST Search is based on the blastall program from Altschul *et al.* [54], searching for regions of similarity and producing ungapped or gapped alignments of these regions between a query sequence and database sequences. The PDB_nr95 database is relatively smaller but collected the protein sequences that have a 3D structure. Proper structures of related proteins were selected as templates. Some SecA protein structures were not included because in these structures large regions that should be contained in SecA^N^ are missing. The selected structures had been loaded from the server before these protein sequences were aligned based on their structure similarities using the Align Structures protocol in the Modeler package. Then these aligned structures and the sequences of SecA^N^/SecA truncation/SecA or their mutants are input to generate their homology models.

### The generated structures of SecA^N^ were evaluated with the Ramachandran plot and the Profiles‐3D

The Ramachandran plot [39] can be used to assess the predicted torsion angles (represented by φ and ψ, which are the torsion angles on either side of the alpha carbons) in proteins, which indicates conformations of the local backbone of each residue in points. The torsion angles of each residue were displayed as points in different colors to distinguish the favorable and unfavorable residues, which were included in the favorable and unfavorable regions, respectively, as represented in the plot. Using this plot, whether the residues are correctively built could be checked.

The Profiles‐3D represents the 3D structure in profile scores related to the residue environments, to assess the compatibility of a sequence with a 3D structure [40]. The sum of the score of each residue is the Verify Score, which can be used to verify the overall quality of the modeled protein structure. The results were displayed in solid ribbon style. The variations in ribbon width and spectrum color were regarded to the Verify Score of each residue. Regions shown in relatively narrower bands and colored blue were more reliable than those shown in broader bands and colored red. The Expected High Score is calculated based on the high‐resolution structures in the PDB, whereas the Expected Low Score is 45% of the Expected High Score. The structure with Verify Score higher than the Expected High Score or between the Expected High and Low Score may be correct. The structure with Verify Score lower than the Expected Low Score would be grossly misfolded.

### Models for the dimer of SecA^N^/SecA truncation/SecA were constructed using the ZDOCK algorithm

The ZDOCK algorithm was developed by Chen and Weng [37] to provide the initial stage of the unbound protein–protein docking between two structures determined by either experiments or modeling. ZDOCK is a rigid‐body docking, using the Pairwise Shape Complementarity method to predict a protein complex, which is based on all close atomic contacts within a specific cutoff distance and usually provides better results than the Grid‐based Shape Complementarity [38]. The obtained poses were clustered according to the position of ligands. The experimentally identified binding site residues could be introduced to filter poses through setting the residues in or outside the binding interface.

3D point plots were made to display the docking results as a 3D plot of points, through which the relationships among properties such as the ZDOCK score, density and cluster of poses could be explored.

The poses can be reranked by the ZRANK scoring program [43] to improve the success rate of prediction for protein complex. ZRANK uses the more detailed energy function [43] but still is quick and accurate enough. Poses with a high density, a high ZDOCK score and a low ZRANK score were then selected.

### Models for the core‐complex composed of the dimer of SecA^N^/SecA truncation/SecA and the BAM complex were constructed using the ZDOCK algorithm

The approaches and process here were similar to those used in building models for the dimer of SecA^N^/SecA truncation/SecA.

## Conflict of interest

The authors declare no conflict of interest.

## Author contributions

FJ designed and performed the modeling, analyzed the data and prepared the manuscript.

## Supporting information


**Fig. S1.** The SecA protein contains multiple domains. The SecA protein contains multiple domains, which are the N‐terminal region of the first nucleotide binding domain (NBD1(N), blue), the peptide binding domain (PPXD, orange), the C‐terminal region of NBD1 (NBD1(C), blue), the second nucleotide binding domain (NBD2, cyan), the HSD (green), the HWD (purple) and the C‐terminal Zn^2+^ binding region (red). The residue position for each domain was indicated in the figure.
**Fig. S2.** The comparison between the theoretical structures for the shortened SecA (SecA^N^/SecA truncation) and the experimentally determined structures for SecA. (A, G, M) Shown are aligning the models of SecA^N^375 (A), SecA^N^416 (G) or SecA596 (M) with SecA structures, including 1TF5 (cyan), 2IPC (blue), 1NKT (light green), 3DIN (pink) and 2VDA (purple). SecA^N^375, SecA^N^416 or SecA596 was colored red. (B–F, H–L, N–R) Shown are aligning the structures of SecA^N^375 (B–F), SecA^N^416 (H–L) or SecA596 (N–R) with 1TF5 (B, H, N), 2IPC (C, I, O), 1NKT (D, J, P), 3DIN (E, K, Q) or 2VDA (F, L, R), respectively.
**Fig. S3.** A representative model for the SecA^N^ dimer in which the GXXXG motif of each monomer was inside the binding interface. (A, B) Shown are the SecA^N^ dimer, in which the two SecA^N^ monomers are colored gray (the receptor, designated as SecA^N^375(R)) and cyan (the ligand, designated as SecA^N^375(L)), respectively. The GXXXG motif was colored red, and the residue 47 was colored red and shown as stick. Binding interface was colored yellow in (B).
**Fig. S4.** Filtered poses for the dimer of SecA^N^/SecA truncation/SecA were displayed with the 3D plot. Plotting the ZDOCK score (*x*) versus the cluster (*y*) versus the density (*z*), the filtered poses for the SecA^N^375 dimer (A), the SecA^N^416 dimer (B), the SecA596 dimer (C) and the SecA dimer (D) were displayed. The poses were colored according to their ZDOCK scores as indicated in the figure.
**Fig. S5.** Filtered poses for the SecA^N^375‐BAM core‐complex were displayed with the 3D plot. Plotting the ZDOCK score (*x*) versus the cluster (*y*) versus the density (*z*), the filtered poses for the core‐complex constructed by docking SecA^N^375‐D53 (A), ‐D20 (C) or ‐D42 (E) to the BAM complex in the ‘close’ conformation (PDB: http://www.rcsb.org/pdb/search/structidSearch.do?structureId=5AYW) and by docking SecA^N^375‐D53 (B), ‐D20 (D) or ‐D42 (F) to the BAM complex in the ‘open’ conformation (PDB: http://www.rcsb.org/pdb/search/structidSearch.do?structureId=5EKQ) were displayed. The poses were colored according to their ZDOCK scores as indicated in the figure.
**Fig. S6.** Filtered poses for the SecA^N^416‐BAM core‐complex were displayed with the 3D plot. Plotting the ZDOCK score (*x*) versus the cluster (*y*) versus the density (*z*), the filtered poses for the core‐complex constructed by docking SecA^N^416‐D20 (A), ‐D25 (C) or ‐D124 (E) to the BAM complex in the ‘close’ conformation (PDB: http://www.rcsb.org/pdb/search/structidSearch.do?structureId=5AYW) and by docking SecA^N^416‐D20 (B), ‐D25 (D) or ‐D124 (F) to the BAM complex in the ‘open’ conformation (PDB: http://www.rcsb.org/pdb/search/structidSearch.do?structureId=5EKQ) were displayed. The poses were colored according to their ZDOCK scores as indicated in the figure.
**Fig. S7.** The representative model for the SecA^N^‐BAM core‐complex in a wrong conformation. Shown was a wrong conformation for the SecA^N^‐BAM core‐complex that resulted from docking SecA^N^375‐D53 to the BAM complex in the ‘close’ conformation (PDB: http://www.rcsb.org/pdb/search/structidSearch.do?structureId=5AYW). The five subunits of BAM were colored dark green (BamA), brown (BamB), purple (BamC), orange (BamD) and red (BamE). SecA^N^375(R) and SecA^N^375(L) in the SecA^N^ dimer were colored dark gray and blue. Dashed lines indicated the position of the OM, the periplasm (periplasmic space) or the IM.
**Fig. S8.** The experimentally determined structure of the *E. coli* SecA dimer (PDB: http://www.rcsb.org/pdb/search/structidSearch.do?structureId=2FSF). (A, B) Shown is the experimentally determined structure of the *E. coli* SecA dimer (PDB: http://www.rcsb.org/pdb/search/structidSearch.do?structureId=2FSF). The two SecA monomers were colored gray and cyan, respectively. The GXXXG motif was colored red, whereas the residue 47 was colored red and shown as stick. The binding interface was marked yellow in (B). The GXXXG motifs were not in the binding interface.
**Fig. S9.** Filtered poses for the SecA596‐BAM core‐complex were displayed with the 3D plot. Plotting the ZDOCK score (*x*) versus the cluster (*y*) versus the density (*z*), the filtered poses for the core‐complex constructed by docking SecA596‐D8 (A), ‐D16 (C) or ‐D68 (E) to the BAM complex in the ‘close’ conformation (PDB: http://www.rcsb.org/pdb/search/structidSearch.do?structureId=5AYW) and by docking SecA596‐D8 (B), ‐D16 (D) or ‐D68 (F) to the BAM complex in the ‘open’ conformation (PDB: http://www.rcsb.org/pdb/search/structidSearch.do?structureId=5EKQ) were displayed. The poses were colored according to their ZDOCK scores as indicated in the figure.
**Fig. S10.** Filtered poses for the SecA‐BAM core‐complex were displayed with the 3D plot. Plotting the ZDOCK score (*x*) versus the cluster (*y*) versus the density (*z*), the filtered poses for the core‐complex constructed by docking SecA‐D141 (A), ‐D114 (C), ‐D91 (E) or 2FSF (G) to the BAM complex in the ‘close’ conformation (PDB: http://www.rcsb.org/pdb/search/structidSearch.do?structureId=5AYW) and by docking SecA‐D141 (B), ‐D114 (D), ‐D91 (F) or 2FSF (H) to the BAM complex in the ‘open’ conformation (PDB: http://www.rcsb.org/pdb/search/structidSearch.do?structureId=5EKQ) were displayed. The poses were colored according to their ZDOCK scores as indicated in the figure.
**Fig. S11**. The predicted structure for the SecA‐associated SecA^N^‐BAM core‐complex. The model for SecA was docked to the model for the SecA^N^375‐BAM core‐complex in either the ‘close’ or the ‘open’ conformation. No proper model was obtained when the core‐complex was in the ‘close’ conformation. However, when the core‐complex was in the ‘open’ conformation, SecA could be docked to the cytoplasm‐exposed C‐terminal regions of SecA^N^375(R) as indicated in the figure. The five subunits of the BAM complex were colored light green (BamA), light purple (BamC), yellow (BamD) and vermilion (BamE). SecA^N^375(R) and SecA^N^375(L) were colored light gray and cyan. SecA was colored purple.
**Table S1.** The main‐chain RMSD and the number of overlapping residues between the listed structures, including the structures of templates and the homology models for SecA^N^/truncation/SecA.
**Table S2.** (A) Generated models for SecA^N^375 sorted by the PDF Total Energy. (B) Generated models for SecA^N^416 sorted by the PDF Total Energy. (C) Generated models for SecA596 sorted by the PDF Total Energy. (D) Generated models for SecA sorted by the PDF Total Energy.
**Table S3.** Verifications of the models for SecA^N^/SecA truncation/SecA with the profiles‐3D.
**Table S4.** (A) The main‐chain RMSD and the number of overlapping residues between the NBD1 (N) domains of the listed SecA and shortened SecA structures. (B) The main‐chain RMSD and the number of overlapping residues between the PPXD domains of the listed SecA and shortened SecA structures. (C) The main‐chain RMSD and the number of overlapping residues between the NBD1(C) domains of the listed SecA and shortened SecA structures. (D) The main‐chain RMSD and the number of overlapping residues between the NBD2 domains of the listed SecA and shortened SecA structures. (E) The main‐chain RMSD and the number of overlapping residues between the C‐terminal regions of the listed SecA and shortened SecA structures.
**Table S5**. Dimers for SecA^N^/SecA truncation/SecA in different conformations were predicted with ZDOCK.
**Table S6.** (A) Optimal models for SecA^N^375 mutants. (B) Optimal models for SecA^N^416 mutants. (C) Optimal models for SecA596 mutants. (D) Optimal models for SecA mutants.
**Table S7.** (A) The main‐chain RMSD and the number of overlapping residues between the models for SecA^N^375 and SecA^N^375 mutants. (B) The main‐chain RMSD and the number of overlapping residues betweenthe models for SecA^N^416 and SecA^N^416 mutants. (C) The main‐chain RMSD and the number of overlapping residues between the models for SecA596 and SecA596 mutants. (D) The main‐chain RMSD and the number of overlapping residues between the models for SecA and SecA mutants.
**Table S8.** (A) Models for the core‐complex composed of SecA^N^375‐D42 and the BAM complex in the ‘close’ conformation. (B) Models for the core‐complex composed of SecA^N^375‐D42 and the BAM complex in the ‘open’ conformation. (C) Models for the core‐complex composed of SecA^N^416‐D20 and the BAM complex in the ‘close’ conformation. (D) Models for the core‐complex composed of SecA^N^416‐D20 and the BAM complex in the ‘open’ conformation.
**Table S9.** Models for the core‐complex composed of SecA596‐D16 and the BAM complex in the ‘open’ conformation.
