# Supplementary material for: Structural insights into the mechanism of a novel protein targeting pathway in Gram‐negative bacteria
Source: FEBS Open Bio. 2020 Mar 9;10(4):561–79. doi: 10.1002/2211-5463.12813 (PMC7137807; doi:10.1002/2211-5463.12813)
Supplement: Supplementary file 1 — Fig. S1. The SecA protein contains multiple domains. The SecA protein contains multiple domains, which are the N‐terminal region of the first nucleotide binding domain (NBD1(N), blue), the peptide binding domain (PPXD, orange), the C‐terminal region of NBD1 (NBD1(C), blue), the second nucleotide binding domain (NBD2, cyan), the HSD (green), the HWD (purple) and the C‐terminal Zn2+ binding region (red). The residue position for each domain was indicated in the figure. Fig. S2. The comparison between the theoretical structures for the shortened SecA (SecAN/SecA truncation) and the experimentally determined structures for SecA. (A, G, M) Shown are aligning the models of SecAN375 (A), SecAN416 (G) or SecA596 (M) with SecA structures, including 1TF5 (cyan), 2IPC (blue), 1NKT (light green), 3DIN (pink) and 2VDA (purple). SecAN375, SecAN416 or SecA596 was colored red. (B–F, H–L, N–R) Shown are aligning the structures of SecAN375 (B–F), SecAN416 (H–L) or SecA596 (N–R) with 1TF5 (B, H, N), 2IPC (C, I, O), 1NKT (D, J, P), 3DIN (E, K, Q) or 2VDA (F, L, R), respectively. Fig. S3. A representative model for the SecAN dimer in which the GXXXG motif of each monomer was inside the binding interface. (A, B) Shown are the SecAN dimer, in which the two SecAN monomers are colored gray (the receptor, designated as SecAN375(R)) and cyan (the ligand, designated as SecAN375(L)), respectively. The GXXXG motif was colored red, and the residue 47 was colored red and shown as stick. Binding interface was colored yellow in (B). Fig. S4. Filtered poses for the dimer of SecAN/SecA truncation/SecA were displayed with the 3D plot. Plotting the ZDOCK score (x) versus the cluster (y) versus the density (z), the filtered poses for the SecAN375 dimer (A), the SecAN416 dimer (B), the SecA596 dimer (C) and the SecA dimer (D) were displayed. The poses were colored according to their ZDOCK scores as indicated in the figure. Fig. S5. Filtered poses for the SecAN375‐BAM core‐complex were displayed with the 3 [file FEB4-10-561-s001.pdf]

Supplementary Materials

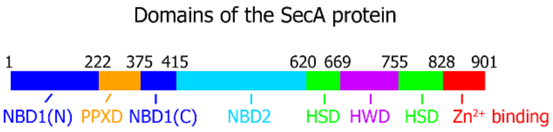

Fig. S1. The SecA protein contains multiple domains. The SecA protein contains multiple domains, which are the N - terminal region of the first nucleotide binding domain (NBD1(N), blue), the peptide binding domain (PPXD, orange), the C - terminal region of NBD1 (NBD1(C), blue), the second nucleotide binding domain (NBD2, cyan), the HSD (green), the HWD (purple) and the C - terminal Zn^{2+} binding region (red). The residue position for each domain was indicated in the figure.

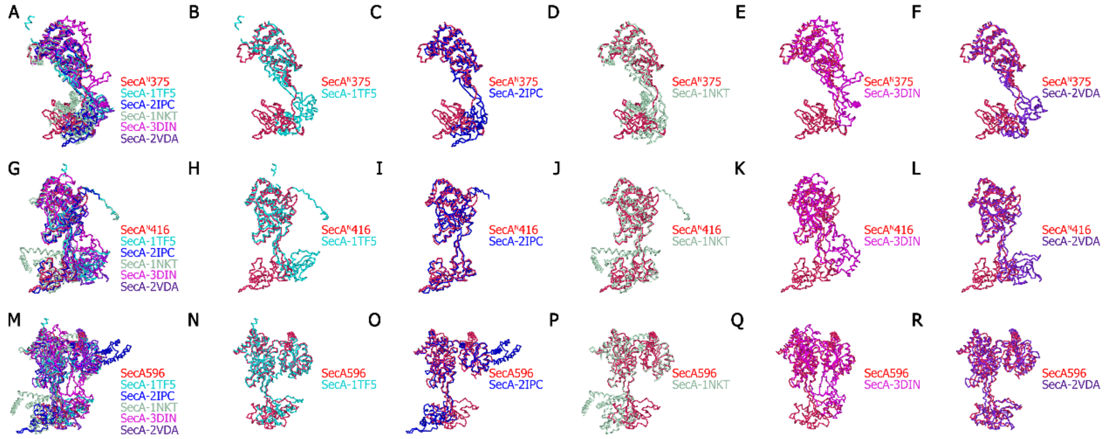

Fig. S2. The comparison between the theoretical structures for the shortened SecA (SecA^{N}/SecA truncation) and the experimentally determined structures for SecA. (A, G, M) Shown are aligning the models of $\text{SecA}^{N}375$ (A), $\text{SecA}^{N}416$ (G) or SecA596 (M) with SecA structures, including 1TF5 (cyan), 2IPC (blue), 1NKT (light green), 3DIN (pink) and 2VDA (purple). $\text{SecA}^{N}375$, $\text{SecA}^{N}416$ or SecA596 was colored red. (B – F, H – L, N – R) Shown are aligning the structures of $\text{SecA}^{N}375$ (B – F), $\text{SecA}^{N}416$ (H – L) or SecA596 (N – R) with 1TF5 (B, H, N), 2IPC (C, I, O), 1NKT (D, J, P), 3DIN (E, K, Q) or 2VDA (F, L, R), respectively.

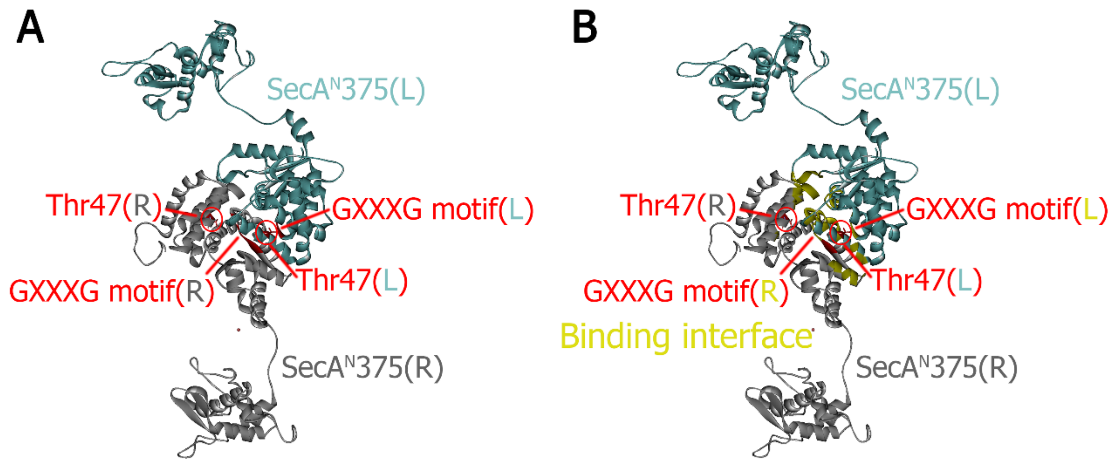

Fig. S3. A representative model for the SecA^{N} dimer in which the GXXXG motif of each monomer was inside the binding interface. (A, B) Shown are the SecA^{N} dimer, in which the two SecA^{N} monomers are colored gray (the receptor, designated as SecA^{N}375(R)) and cyan (the ligand, designated as SecA^{N}375(L)), respectively. The GXXXG motif was colored red, and the residue 47 was colored red and shown as stick. Binding interface was colored yellow in (B).

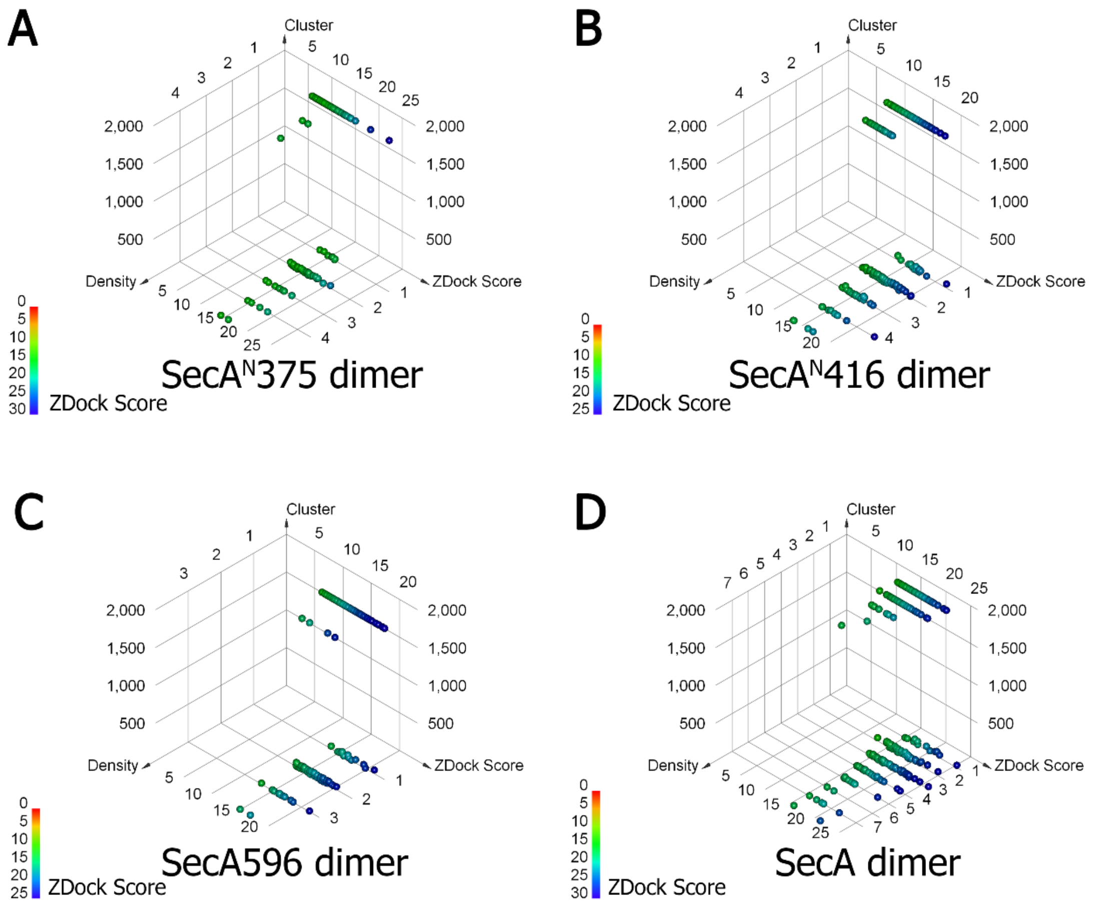

Fig. S4. Filtered poses for the dimer of SecA^{N}/SecA truncation/SecA were displayed with the 3D plot. Plotting the ZDOCK score (*x*) versus the cluster (*y*) versus the density (*z*), the filtered poses for the SecA^{N}375 dimer (A), the SecA^{N}416 dimer (B), the SecA596 dimer (C) and the SecA dimer (D) were displayed. The poses were colored according to their ZDOCK scores as indicated in the figure.

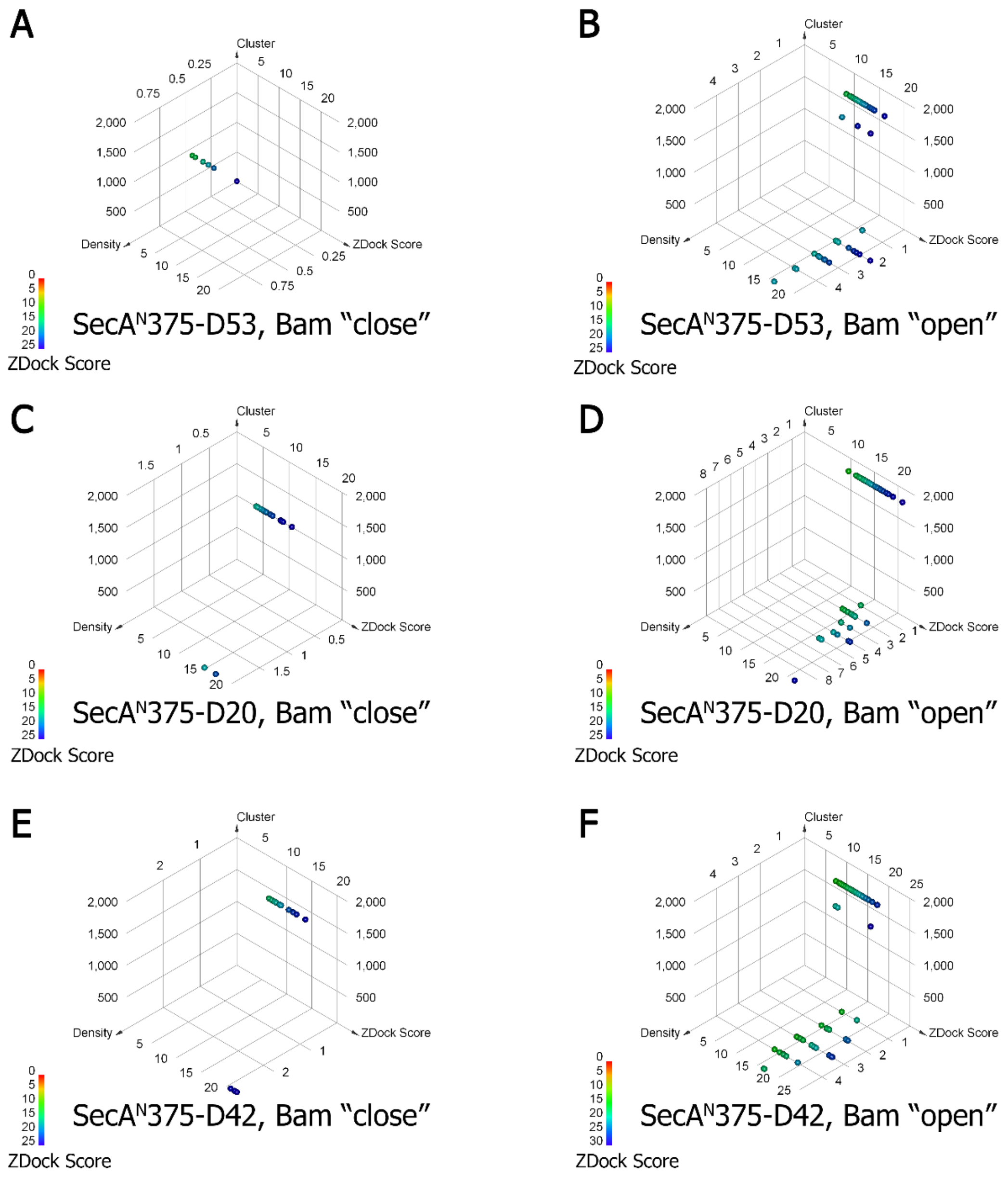

Fig. S5. Filtered poses for the SecA^{N}375 - BAM core - complex were displayed with the 3D plot. Plotting the ZDOCK score (*x*) versus the cluster (*y*) versus the density (*z*), the filtered poses for the core - complex constructed by docking SecA^{N}375 - D53 (A), - D20 (C) or - D42 (E) to the BAM complex in the 'close' conformation (PDB: 5AYW) and by docking SecA^{N}375 - D53 (B), - D20 (D) or - D42 (F) to the BAM complex in the 'open' conformation (PDB: 5EKQ) were displayed. The poses were colored according to their ZDOCK scores as indicated in the figure.

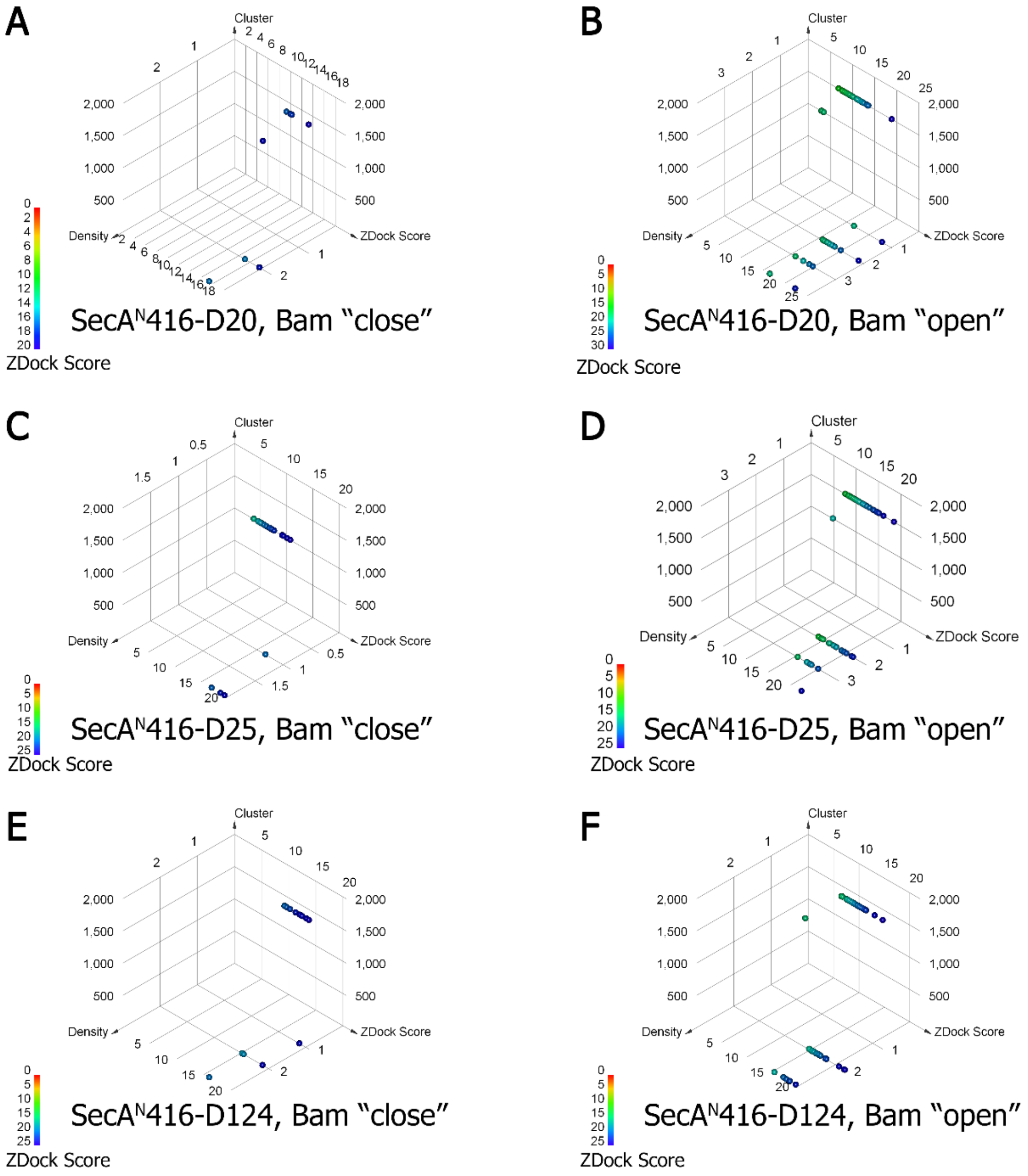

Fig. S6. Filtered poses for the SecA^{N}416 - BAM core - complex were displayed with the 3D plot. Plotting the ZDOCK score (*x*) versus the cluster (*y*) versus the density (*z*), the filtered poses for the core - complex constructed by docking SecA^{N}416 - D20 (A), - D25 (C) or - D124 (E) to the BAM complex in the 'close' conformation (PDB: 5AYW) and by docking SecA^{N}416 - D20 (B), - D25 (D) or - D124 (F) to the BAM complex in the 'open' conformation (PDB: 5EKQ) were displayed. The poses were colored according to their ZDOCK scores as indicated in the figure.

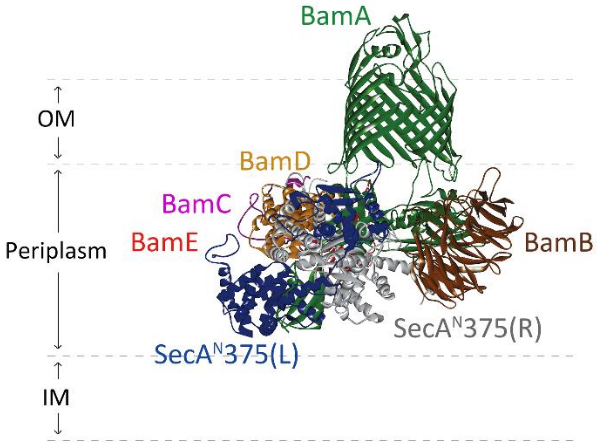

Fig. S7. The representative model for the SecA^{N} - BAM core - complex in a wrong conformation. Shown was a wrong conformation for the SecA^{N} - BAM core - complex that resulted from docking SecA^{N}375 - D53 to the BAM complex in the 'close' conformation (PDB: 5AYW). The five subunits of BAM were colored dark green (BamA), brown (BamB), purple (BamC), orange (BamD) and red (BamE). SecA^{N}375(R) and SecA^{N}375(L) in the SecA^{N} dimer were colored dark gray and blue. Dashed lines indicated the position of the OM, the periplasm (periplasmic space) or the IM.

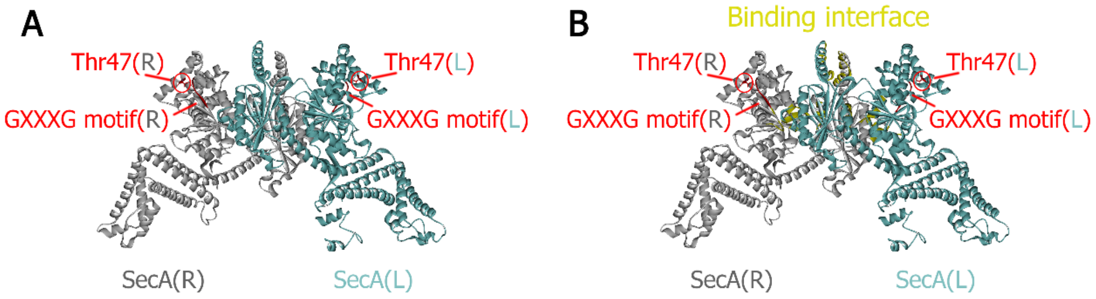

Fig. S8. The experimentally determined structure of the *E. coli* SecA dimer (PDB: 2FSF). (A, B) Shown is the experimentally determined structure of the *E. coli* SecA dimer (PDB: 2FSF). The two SecA monomers were colored gray and cyan, respectively. The GXXXG motif was colored red, whereas the residue 47 was colored red and shown as stick. The binding interface was marked yellow in (B). The GXXXG motifs were not in the binding interface.

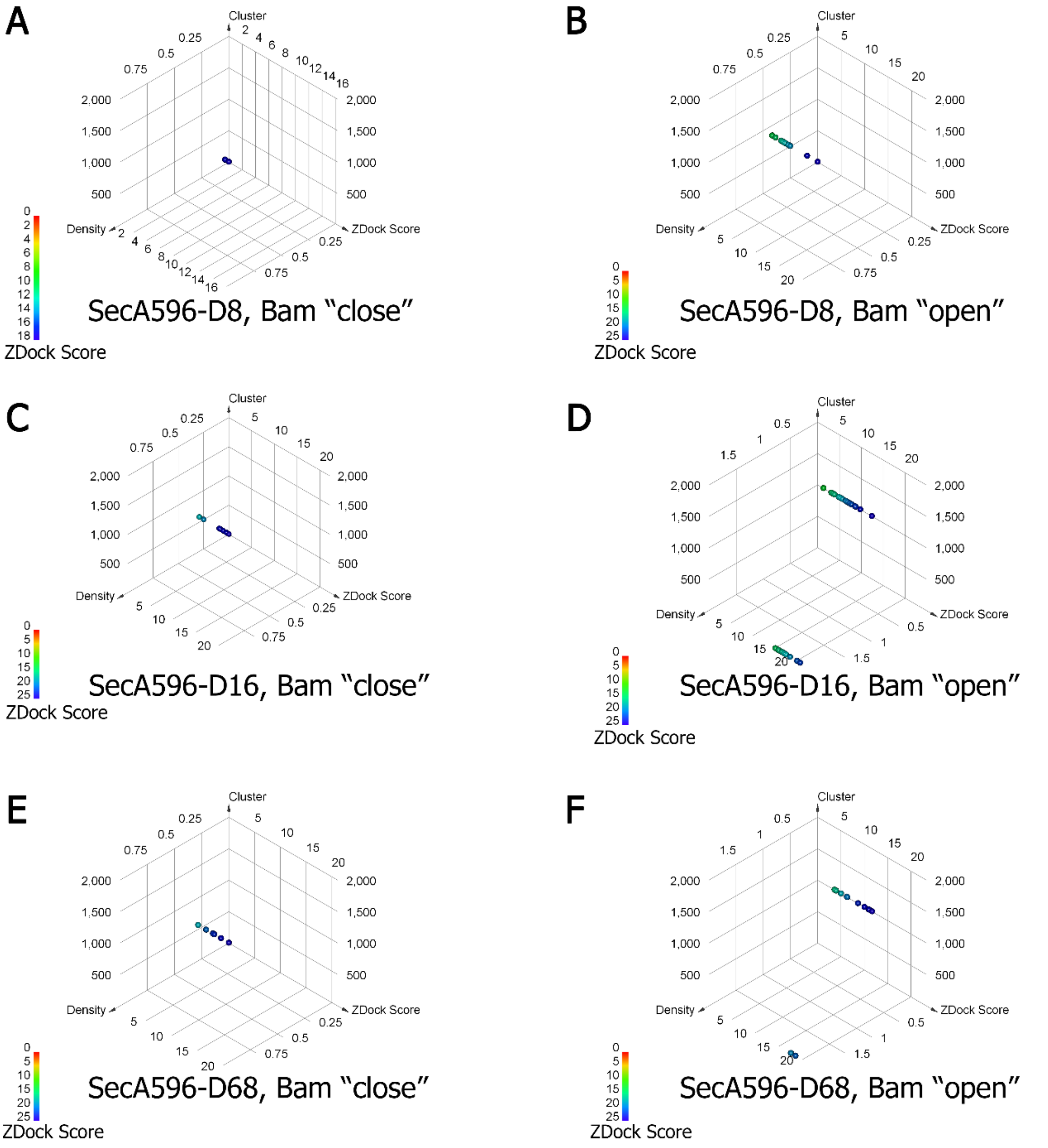

Fig. S9. Filtered poses for the SecA596 - BAM core - complex were displayed with the 3D plot. Plotting the ZDOCK score (*x*) versus the cluster (*y*) versus the density (*z*), the filtered poses for the core - complex constructed by docking SecA596 - D8 (A), - D16 (C) or - D68 (E) to the BAM complex in the 'close' conformation (PDB: 5AYW) and by docking SecA596 - D8 (B), - D16 (D) or - D68 (F) to the BAM complex in the 'open' conformation (PDB: 5EKQ) were displayed. The poses were colored according to their ZDOCK scores as indicated in the figure.

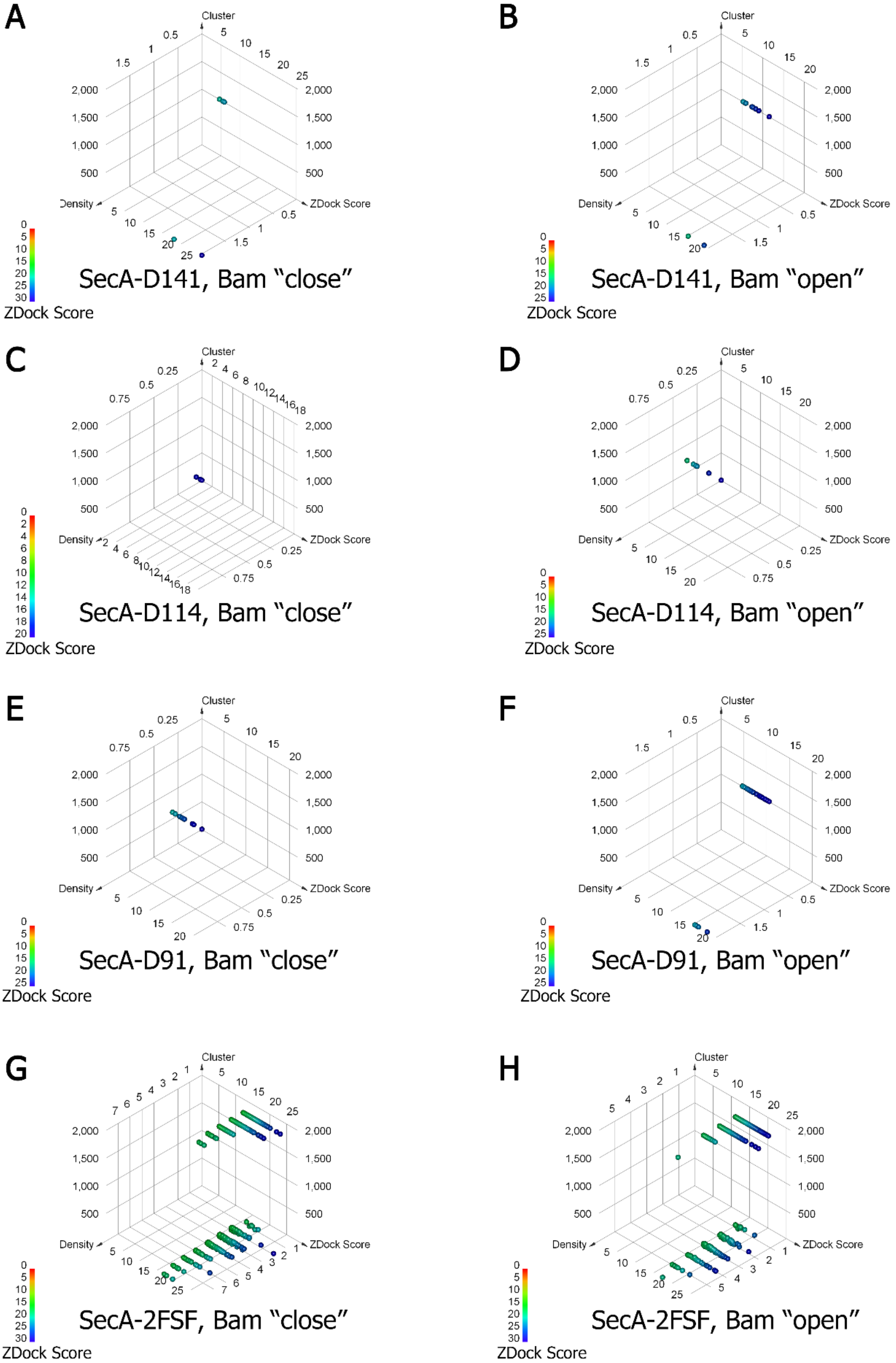

Fig. S10. Filtered poses for the SecA - BAM core - complex were displayed with the 3D plot. Plotting the ZDOCK score (*x*) versus the cluster (*y*) versus the density (*z*), the filtered poses for

the core - complex constructed by docking SecA - D141 (A), - D114 (C), - D91 (E) or 2FSF (G) to the BAM complex in the 'close' conformation (PDB: 5AYW) and by docking SecA - D141 (B), - D114 (D), - D91 (F) or 2FSF (H) to the BAM complex in the 'open' conformation (PDB: 5EKQ) were displayed. The poses were colored according to their ZDOCK scores as indicated in the figure.

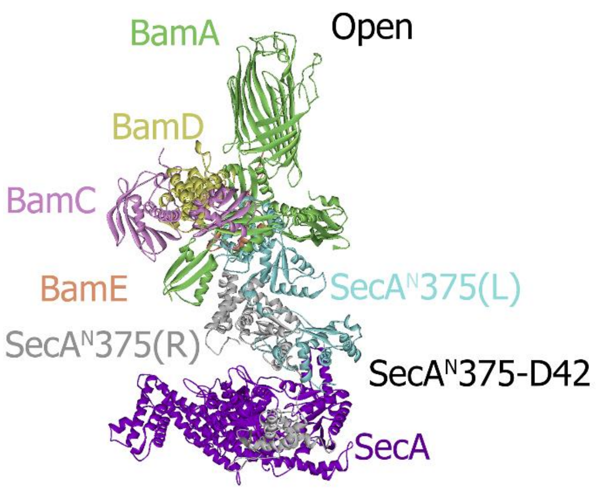

Fig. S11. The predicted structure for the SecA - associated SecA^N - BAM core - complex. The model for SecA was docked to the model for the SecA^{N}375 - BAM core - complex in either the 'close' or the 'open' conformation. No proper model was obtained when the core - complex was in the 'close' conformation. However, when the core - complex was in the 'open' conformation, SecA could be docked to the cytoplasm - exposed C - terminal regions of SecA^{N}375(R) as indicated in the figure. The five subunits of the BAM complex were colored light green (BamA), light purple (BamC), yellow (BamD) and vermilion (BamE). SecA^{N}375(R) and SecA^{N}375(L) were colored light gray and cyan. SecA was colored purple.

Table S1. The main - chain RMSD and the number of overlapping residues between the listed structures, including the structures of templates and the homology models for $SecA^N$/truncation/SecA.

	1TF5	2IPC	1NKT	3DIN	1T3N	2QDE	1P0K	1UXA	2G50	2VDA	$SecA^N375$	$SecA^N416$	SecA596	SecA
**1TF5**		662	660	638	112	59	90	44	99	741	214	292	530	749
**2IPC**	3.404Å		791	629	114	66	93	35	102	710	228	405	483	725
**1NKT**	3.12Å	2.547Å		636	107	70	90	35	99	696	228	384	483	716
**3DIN**	3.398Å	3.985Å	3.814Å		95	59	96	48	101	638	214	270	437	647
**1T3N**	5.879Å	5.808Å	5.894Å	6.116Å		57	81	12	79	114	58	74	107	116
**2QDE**	5.855Å	5.965Å	6.23Å	6.474Å	6.705Å		58	13	59	64	43	53	61	61
**1P0K**	6.465Å	6.693Å	6.575Å	6.189Å	6.688Å	6.208Å		4	187	90	54	54	67	62
**1UXA**	4.972Å	5.254Å	5.082Å	5.284Å	7.13Å	7.092Å	6.591Å		16	44	11	28	37	61
**2G50**	5.643Å	6.161Å	6.088Å	5.738Å	6.297Å	6.646Å	4.954Å	4.212Å		97	63	63	86	60
**2VDA**	4.081Å	4.265Å	4.127Å	5.132Å	5.983Å	5.626Å	6.417Å	5.569Å	6.449Å		215	304	564	809
**$SecA^N375$**	1.398Å	3.687Å	3.774Å	2.605Å	6.05Å	6.543Å	5.953Å	5.344Å	5.309Å	1.865Å		232	220	226
**$SecA^N416$**	3.652Å	3.461Å	3.958Å	2.754Å	6.026Å	6.049Å	6.323Å	5.088Å	5.668Å	3.6Å	2.881Å		308	306
**SecA596**	2.87Å	2.815Å	2.928Å	3.452Å	5.727Å	6.046Å	6.621Å	4.976Å	5.826Å	3.363Å	2.138Å	3.183Å		575
**SecA**	2.822Å	3.659Å	3.478Å	3.425Å	5.785Å	4.84Å	5.483Å	5.472Å	5.185Å	3.224Å	1.96Å	2.637Å	2.558Å	

Table S2A. Generated models for $SecA^N375$ sorted by the PDF Total Energy.

Model Name	PDF Total Energy	PDF Physical Energy	DOPE Score
$SecA^N375\_02$	15427.36	971.7	-35181.26
$SecA^N375\_05$	15549.75	960.17	-36477.13
$SecA^N375\_01$	15646.55	989.24	-36140.65
$SecA^N375\_04$	16088.22	1336.18	-34720.32
$SecA^N375\_03$	16840.07	1165.13	-33852.89

Table S2B. Generated models for $SecA^N416$ sorted by the PDF Total Energy.

Model Name	PDF Total Energy	PDF Physical Energy	DOPE Score
$SecA^N416\_02$	18957.9	1243.12	-42103.39
$SecA^N416\_05$	19206.01	1308.51	-42860.64
$SecA^N416\_01$	19206.7	1290.81	-42346.59
$SecA^N416\_03$	19304.01	1272.49	-42403.42
$SecA^N416\_04$	19427.33	1453.66	-41895.01

Table S2C. Generated models for SecA596 sorted by the PDF Total Energy.

Model Name	PDF Total Energy	PDF Physical Energy	DOPE Score
SecA596_01	27042.5	1729.91	-60972.5
SecA596_03	27401.6	1625.74	-60245.23
SecA596_05	27448.45	1684.37	-60408.44
SecA596_04	27523.1	1667.06	-60052.18
SecA596_02	27813.7	1735.83	-59137.45

Table S2D. Generated models for SecA sorted by the PDF Total Energy.

Model Name	PDF Total Energy	PDF Physical Energy	DOPE Score
SecA_05	46145.58	2621.35	-91107.14
SecA_03	46532.64	2796.24	-90068.85
SecA_01	46616.41	2765.21	-88637.21
SecA_02	46790.21	2955.58	-87233.32
SecA_04	47466.01	3046.76	-87435.76

Table S3. Verifications of the models for $SecA^N$/SecA truncation/SecA with the profiles - 3D.

	$SecA^N$375	$SecA^N$416	SecA595	SecA
Verify high score	170.945	189.792	272.697	406.674
Verify score	103.43	149.92	211.64	334.71
Verify low score	76.9252	85.4066	122.714	183.003

Table S4A. The main - chain RMSD and the number of overlapping residues between the NBD1 (N) domains of the listed SecA and shortened SecA structures.

	$SecA^N$375	$SecA^N$416	SecA596	SecA	2VDA	2FSF
$SecA^N$375		215	218	218	215	215
$SecA^N$416	1.084 Å		216	216	217	216
SecA596	0.964 Å	0.654 Å		227	216	216
SecA	1.6 Å	1.266 Å	1.537 Å		216	216
2VDA	1.098 Å	0.799 Å	0.855 Å	1.455 Å		216
2FSF	0.939 Å	0.727 Å	0.867 Å	1.455 Å	0.725 Å	

Table S4B. The main - chain RMSD and the number of overlapping residues between the PPXD domains of the listed SecA and shortened SecA structures.

	$SecA^{N}375$	$SecA^{N}416$	SecA596	SecA	2VDA
**$SecA^{N}375$**		136	121	108	123
**$SecA^{N}416$**	3.731 Å		132	119	134
**SecA596**	3.971 Å	3.833 Å		124	143
**SecA**	4.144 Å	4.102 Å	1.873 Å		120
**2VDA**	4.007 Å	3.913 Å	1.337 Å	2.068 Å	

Table S4C. The main - chain RMSD and the number of overlapping residues between the NBD1(C) domains of the listed SecA and shortened SecA structures.

	$SecA^{N}416$	SecA596	SecA	2VDA	2FSF
**$SecA^{N}416$**		41	41	41	41
**SecA596**	0.563 Å		41	41	41
**SecA**	1.238 Å	1.367 Å		41	41
**2VDA**	1.362 Å	1.473 Å	0.648 Å		41
**2FSF**	1.316 Å	1.405 Å	0.935 Å	0.889 Å	

Table S4D. The main - chain RMSD and the number of overlapping residues between the NBD2 domains of the listed SecA and shortened SecA structures.

	SecA596	SecA	2VDA	2FSF
**SecA596**		180	180	180
**SecA**	0.646 Å		180	180
**2VDA**	1.965 Å	2.09 Å		180
**2FSF**	2.007 Å	2.164 Å	0.801 Å	

Table S4E. The main - chain RMSD and the number of overlapping residues between the C - terminal regions of the listed SecA and shortened SecA structures.

	SecA	2VDA	2FSF
**SecA**		239	237
**2VDA**	2.727 Å		238
**2FSF**	2.738 Å	0.834 Å	

Table S5. Dimers for SecA^N/SecA truncation/SecA in different conformations were predicted with ZDOCK.

Model name	Density	Cluster	ZDOCK Score	ZRANK Score	Conformation
SecA^{N}375-D53	4	2	15.24	-35.587	"T" type
SecA^{N}375-D20	2	8	15.5	-56.151	"T" type
SecA^{N}375-D42	2	12	15.02	-40.796	Parallel
SecA^{N}416-D20	4	2	23.14	-55.551	"X" type
SecA^{N}416-D25	4	3	16.3	-53.469	Head-to-tail
SecA^{N}416-D124	4	4	15.02	-13.586	"7" type
SecA596-D8	3	3	18.56	-67.523	Parallel
SecA596-D16	3	4	16.36	-57.74	Head-to-head
SecA596-D68	3	6	16.78	-33.754	Head-to-head
SecA-D141	8	1	21.1	-2.104	Anti-parallel
SecA-D114	5	-	15.48	-10.558	Head to head
SecA-D91	4	22	19.36	-19.469	"X" type

Table S6A. Optimal models for SecA^{N}375 mutants.

Mutation	PDF Total Energy	PDF Physical Energy	DOPE Score	Verify Score	Potential Energy (kcal/mol)
G151F	15890.02	1014.97	-35948.33	117.07	-6620.24589
L152K	15577.08	1043.87	-35261.83	111.47	-4428.95544
T153K	15585.73	1033.53	-34921.44	115.97	-6706.61712
V154K	15854.15	1028.29	-35192.34	111.78	-5601.18167
G155F	16078.35	1025.91	-35748.13	115.81	-5973.11849

Table S6B. Optimal models for SecA^{N}416 mutants.

Mutation	PDF Total Energy	PDF Physical Energy	DOPE Score	Verify Score	Potential Energy (kcal/mol)
G151F	20880.57	1353.66	-41996.4	141.5	-5973.75947
L152K	20836.79	1297.37	-40883.74	128.72	-5579.18656
T153K	20706.32	1369.41	-41192.53	144.08	-6025.09896
V154K	20858.96	1407.35	-40776.74	140.9	-5836.28588
G155F	21049.9	1362.11	-41863.62	143.96	-5381.72999

Table S6C. Optimal models for SecA596 mutants.

Mutation	PDF Total Energy	PDF Physical Energy	DOPE Score	Verify Score	Potential Energy (kcal/mol)
G151F	27244.12	1545.85	-62157.48	210.2	-10812.01673
L152K	27299.1	1569.9	-61732.12	214.32	-9786.27384
T153K	27484.54	1604.03	-61952.73	227.09	-10427.44487
V154K	27165.74	1543.16	-62114.57	216.61	-11772.18657
G155F	27341.77	1539.37	-61952.62	216.62	-10568.51420

Table S6D. Optimal models for SecA mutants.

Mutation	PDF Total Energy	PDF Physical Energy	DOPE Score	Verify Score	Potential Energy (kcal/mol)
G151F	46284.8	2891.56	-90769.53	309.03	309518.83192
L152K	45959.58	2512.34	-91056.4	312.62	-12886.21854
T153K	46888.92	3032.23	-88411.07	310.34	-11708.19941
V154K	46461.98	2904.67	-90255.59	321.33	-14786.18747
G155F	45439.76	2696.07	-91513.08	315.77	-14966.12769

Table S7A. The main - chain RMSD and the number of overlapping residues between the models for SecA^{N}375 and SecA^{N}375 mutants.

	SecA^{N}375	G151F	L152K	T153K	V154K	G155F
SecA^{N}375		239	277	206	344	234
G151F	2.093 Å		200	203	221	207
L152K	5.907 Å	4.692 Å		227	289	221
T153K	2.772 Å	2.973 Å	4.704 Å		218	217
V154K	4.512 Å	4.759 Å	3.679 Å	4.027 Å		238
G155F	4.129 Å	3.354 Å	2.762 Å	3.056 Å	2.829 Å	

Table S7B. The main - chain RMSD and the number of overlapping residues between the models for SecA^{N}416 and SecA^{N}416 mutants.

	SecA^{N}416	G151F	L152K	T153K	V154K	G155F
SecA^{N}416		313	313	313	313	318
G151F	2.883 Å		414	414	410	407
L152K	2.487 Å	2.687 Å		416	412	405
T153K	2.459 Å	2.683 Å	2.23 Å		412	405
V154K	2.755 Å	1.586 Å	1.613 Å	2.619 Å		401
G155F	2.781 Å	3.299 Å	4.068 Å	3.455 Å	3.967 Å	

Table S7C. The main - chain RMSD and the number of overlapping residues between the models for SecA596 and SecA596 mutants.

	SecA596	G151F	L152K	T153K	V154K	G155F
SecA596		592	592	525	525	592
G151F	1.823 Å		596	527	527	596
L152K	1.751 Å	1.307 Å		527	527	596
T153K	2.523 Å	3.011 Å	3.134 Å		596	527
V154K	2.574 Å	2.879 Å	2.917 Å	1.997 Å		527
G155F	2.046 Å	2.002 Å	2.806 Å	2.855 Å	3.209 Å	

Table S7D. The main - chain RMSD and the number of overlapping residues between the models for SecA and SecA mutants.

	SecA	G151F	L152K	T153K	V154K	G155F
SecA		886	886	877	879	884
G151F	1.486 Å		886	877	879	884
L152K	1.458 Å	1.271 Å		877	879	884
T153K	1.348 Å	1.474 Å	1.605 Å		877	875
V154K	1.602 Å	1.834 Å	1.628 Å	1.727 Å		877
G155F	2.192 Å	2.298 Å	2.202 Å	1.981 Å	2.544 Å	

Table S8A. Models for the core - complex composed of $SecA^{N}375$ - D42 and the BAM complex in the ‘close’ conformation.

	121	129	47	Density	Cluster	ZDOCK Score	Conformation
1		Y		3	1	22.4	
2		Y	Y	3	1	21.58	
4		Y	Y	3	1	20.8	

Table S8B. Models for the core - complex composed of $SecA^{N}375$ - D42 and the BAM complex in the 'open' conformation

	121	129	47	Density	Cluster	ZDOCK Score	Conformation
40		Y		5	1	16.6	
44				5	2	16.48	
14				4	5	19.28	
41				4	2	16.6	
60				4	2	15.9	
3				3	3	22.22	
4		Y		3	4	22.02	
5				3	4	21.5	
18			Y	3	1	18.38	
28	Y	Y		3	1	17.8	
33				3	5	17.32	
66		Y		3	6	15.32	
1		Y		2	2	26.12	
7	Y			2	7	20.74	
9				2	3	20.34	
10		Y		2	8	20.24	
20				2	2	18.3	
27		Y		2	2	17.8	
48				2	9	16.32	
52		Y		2	6	16.18	
56		Y		2	9	16.14	
61				2	3	15.86	
62				2	7	15.86	
63				2	4	15.66	

Table S8C. Models for the core - complex composed of $SecA^{N}416$ - D20 and the BAM complex in the 'close' conformation.

	121	129	47	Density	Cluster	ZDOCK Score	Conformation
9		Y	Y	3	1	15.34	
2		Y	Y	2	2	18.3	
3		Y	Y	2	2	17.72	
4		Y	Y	2	1	17.7	
5		Y		2	2	17.66	W
10		Y		2	1	15.12	

Table S8D. Models for the core - complex composed of SecA^{N}416 - D20 and the BAM complex in the 'open' conformation.

	121	129	47	Density	Cluster	ZDOCK Score	Conformation
13			Y	4	1	17.58	
2		Y		3	2	22.04	W
4				3	1	21.28	W
6			Y	3	2	19.7	W
20		Y		3	3	17.12	W
27		Y		3	1	16.54	
3			Y	2	4	21.8	W
7		Y		2	2	19.54	W
8		Y		2	3	18.96	W
10		Y		2	5	18.16	
14				2	6	17.56	
15				2	7	17.5	W
18		Y		2	8	17.22	W
19		Y		2	5	17.12	
23				2	9	16.82	W
24		Y		2	1	16.74	
26				2	6	16.58	
28		Y	Y	2	2	16.42	
34		Y		2	8	15.76	W
41				2	9	15.22	W
42	Y			2	4	15.16	

Table S9. Models for the core - complex composed of SecA596 - D16 and the BAM complex in the 'open' conformation.

	121	129	47	Density	Cluster	ZDOCK Score	Conformation
7				2	1	20.28	W
35		Y		2	2	15.38	
13		Y		2	2	18.6	
18			Y	2	1	17.32	W
29			Y	2	4	16.14	
14	Y	Y	Y	2	3	18.44	
23		Y	Y	2	3	16.76	
30				2	4	15.98	
